# Immediate newborn care and breastfeeding: EN-BIRTH multi-country validation study

**DOI:** 10.1186/s12884-020-03421-w

**Published:** 2021-03-26

**Authors:** Tazeen Tahsina, Aniqa Tasnim Hossain, Harriet Ruysen, Ahmed Ehsanur Rahman, Louise T. Day, Kimberly Peven, Qazi Sadeq-ur Rahman, Jasmin Khan, Josephine Shabani, Ashish KC, Tapas Mazumder, Sojib Bin Zaman, Shafiqul Ameen, Stefanie Kong, Agbessi Amouzou, Ornella Lincetto, Shams El Arifeen, Joy E. Lawn, EN-BIRTH Study Group, Qazi Sadeq-ur Rahman, Ahmed Ehsanur Rahman, Tazeen Tahsina, Sojib Bin Zaman, Shafiqul Ameen, Tanvir Hossain, Abu Bakkar Siddique, Aniqa Tasnim Hossain, Tapas Mazumder, Jasmin Khan, Taqbir Us Samad Talha, Rajib Haider, Hafizur Rahman, Anisuddin Ahmed, Shams Arifeen, Omkar Basnet, Avinash K. Sunny, Nishant Thakur, Regina Gurung, Anjani Kumar Jha, Bijay Jha, Ram Chandra Bastola, Rajendra Paudel, Asmita Paudel, K. C. Ashish, Nahya Salim, Donat Shamba, Josephine Shabani, Kizito Shirima, Menna Narcis Tarimo, Godfrey Mbaruku, Honorati Masanja, Louise T. Day, Harriet Ruysen, Kimberly Peven, Vladimir Sergeevich Gordeev, Georgia R. Gore-Langton, Dorothy Boggs, Stefanie Kong, Angela Baschieri, Simon Cousens, Joy E. Lawn

**Affiliations:** 1 Maternal and Child Health Division, International Centre for Diarrhoeal Disease Research Bangladesh, (icddr,b), 68 Shahid Tajuddin Ahmed Sarani, Mohakhali, Dhaka, Bangladesh; 2 Maternal, Adolescent, Reproductive & Child Health (MARCH) Centre, London School of Hygiene & Tropical Medicine, London, UK; 3 Florence Nightingale Faculty of Nursing, Midwifery & Palliative Care, King’s College London, London, UK; 4 Department of Health Systems, Impact Evaluation and Policy, Ifakara Health Institute (IHI), Dar Es Salaam, Tanzania; 5 Department of Women’s and Children’s Health, International Maternal and Child Health, Uppsala University, Uppsala, Sweden; 6 Johns Hopkins University, Bloomberg School of Public Health, Baltimore, USA; 7 World Health Organization, Geneva, Switzerland

**Keywords:** Birth, Maternal, Newborn, Validity, Survey, Hospital records, Health management systems, Immediate newborn care, Breastfeeding, Skin-to-skin

## Abstract

**Background:**

Immediate newborn care (INC) practices, notably early initiation of breastfeeding (EIBF), are fundamental for newborn health. However, coverage tracking currently relies on household survey data in many settings. “*Every Newborn* Birth Indicators Research Tracking in Hospitals” (EN-BIRTH) was an observational study validating selected maternal and newborn health indicators. This paper reports results for EIBF.

**Methods:**

The EN-BIRTH study was conducted in five public hospitals in Bangladesh, Nepal, and Tanzania, from July 2017 to July 2018. Clinical observers collected tablet-based, time-stamped data on EIBF and INC practices (skin-to-skin within 1 h of birth, drying, and delayed cord clamping). To assess validity of EIBF measurement, we compared observation as gold standard to register records and women’s exit-interview survey reports. Percent agreement was used to assess agreement between EIBF and INC practices. Kaplan Meier survival curves showed timing. Qualitative interviews were conducted to explore barriers/enablers to register recording.

**Results:**

Coverage of EIBF among 7802 newborns observed for ≥1 h was low (10.9, 95% CI 3.8–21.0). Survey-reported (53.2, 95% CI 39.4–66.8) and register-recorded results (85.9, 95% CI 58.1–99.6) overestimated coverage compared to observed levels across all hospitals. Registers did not capture other INC practices apart from breastfeeding. Agreement of EIBF with other INC practices was high for skin-to-skin (69.5–93.9%) at four sites, but fair/poor for delayed cord-clamping (47.3–73.5%) and drying (7.3–29.0%). EIBF and skin-to-skin were the most delayed and EIBF rarely happened after caesarean section (0.5–3.6%). Qualitative findings suggested that focusing on accuracy, as well as completeness, contributes to higher quality with register reporting.

**Conclusions:**

Our study highlights the importance of tracking EIBF despite measurement challenges and found low coverage levels, particularly after caesarean births. Both survey-reported and register-recorded data over-estimated coverage. EIBF had a strong agreement with skin-to-skin but is not a simple tracer for other INC indicators. Other INC practices are challenging to measure in surveys, not included in registers, and are likely to require special studies or audits. Continued focus on EIBF is crucial to inform efforts to improve provider practices and increase coverage. Investment and innovation are required to improve measurement.

**Supplementary Information:**

The online version contains supplementary material available at 10.1186/s12884-020-03421-w.

## Key findings

**Table:** 

**What is known and what is new about this study?** • Breastfeeding has strong evidence of high impact on child mortality and morbidity, is a core indicator for child health and nutrition, and is already measured in nationally representative household surveys. • Challenges exist for measurement of breastfeeding and other immediate newborn care (INC) practices such as skin-to-skin, drying and cord care in many high mortality settings where most data are collected via household surveys conducted every 2–5 years, although around three-quarters of births globally now occur in facilities. Routine data may have utility for providing more timely data on INC practices. However, there are limited studies comparing observed EIBF with both register and survey data, or exploring if EIBF can be used as a tracer for other INC practices. • The EN-BIRTH study in Bangladesh, Nepal, and Tanzania included > 23,000 births, with 7802 newborns observed for at least 1 h after birth, and is the largest indicator validation study to date. Observations were time-stamped, and our large sample size enabled examination of timing of early initiation of breastfeeding within 1 h of birth (EIBF) and newborn care practices, as well as variation between vaginal and caesarean births.	
**Measurement of early initiation of breastfeeding- what did we find?** • Observer-assessed coverage of EIBF was low (10.9%) in these hospitals, particularly after caesarean birth (3.6%). Exit survey-reported coverage of EIBF (‘put to breast’) was 53.2%. Register-recorded coverage overestimated observer-assessed coverage of EIBF in four sites (88.6%). One site (Pokhara, Nepal) had no column regarding breastfeeding. No other INC practices were recorded in registers. Qualitative data suggested that register-recording can be improved with streamlined data collection systems that reduce the workload for frontline staff.	
**Association with other INC practices- what did we find?** • Within observer-assessed data, EIBF had high percentage agreement with skin-to-skin within 1 h of birth in four facilities (70.3–93.9%), and with delayed cord clamping in three facilities (64.6–73.5%). Coverage of immediate drying was very high (~ 99%), early breastfeeding was very low (10.9%), and agreement between these indicators was poor (< 29% in all hospitals).	
**Timing of breastfeeding and INC practices: what did we find?** • Observer-assessed drying (median 0.83 min) and delayed cord clamping (median 1.88 min) were provided rapidly after birth for almost all newborns. EIBF coverage was low, and median time to initiation was > 1 h for all five facilities and markedly delayed for caesarean births.	
**What next and research gaps?** • We recommend renewed focus on improving nationally representative, reliable measurement of EIBF. Survey questions to assess steps (put to breast/attachment/sucking) in the breastfeeding process should be considered, and questionnaires could be adapted with less focus on a rigid time interval to see if this increases accuracy. • Other INC practices are important but are more complex to track in surveys and routine registers; these could be assessed via audits or specific studies. • Root-cause analysis could help identify why certain facilities perform better in providing timely care and help improve practice. These data are needed to inform both health care provider practices and health system actions to address gaps. • Implementation research on register design, implementation, and data flow into health management information systems is also required.	

## Background

Almost half of all deaths in children under the age of five occur in the first month of life (neonatal period), totalling 2.4 million deaths, with 1 million dying on their birthday [1–4]. Most can be prevented with high quality maternal and newborn care, including provision of immediate newborn care (INC) practices as prioritised by the World Health Organization (WHO) [5].

INC practices include skin-to-skin contact during the first hour of life, immediate drying, delayed cord clamping (1–3 min after birth), and early initiation of breastfeeding within 1 h of birth (EIBF) [5]. EIBF has high-quality evidence regarding impact on improving neonatal and under-five mortality and morbidity [6–8], and for improved long-term growth and child development outcomes [9–13]. Delayed cord clamping is also supported by high-quality evidence, and while there are no proven mortality gains, health benefits include lower rates of anaemia [14, 15]. Outcome measures for skin-to-skin and immediate drying often focus on short-term hypothermia reduction (excluding premature babies) [5]. However, the benefits from skin-to-skin care include the promotion of breastfeeding initiation and bonding between mother and child with potential for improved cardiovascular system stability, although evidence is largely observational [12, 16–18]. As such, WHO issued a “strong” recommendation for early skin-to-skin contact as soon after birth as possible for all clinically stable neonates [17, 19].

Population-based surveys, such as the Demographic and Health Survey (DHS) and Multiple Indicator Cluster Surveys (MICS) are the main source of coverage data for INC practices in low- and middle-income countries (LMICs). These are undertaken every 2 to 5 years in about 60 countries. Currently, core questionnaires for both DHS and MICS include questions to capture EIBF and skin-to-skin initiation. Other components of immediate and essential newborn care (such as drying) are in an optional module specific to newborn care [20] (Additional file 1). Of five studies assessing validity of breastfeeding measures using women’s report in survey, three met the criteria for individual validity analyses [21–23]; overall accuracy of breastfeeding in survey-report was inconsistent (Additional File 2) [21–25]. A similar pattern is seen for women’s report of skin-to-skin initiation [21, 25] and immediate drying [21, 23–25]. Collection of accurate survey data around the time of birth is challenging due to recall biases of women particularly regarding interventions provided around the time of birth when multiple events are happening simultaneously; pain and/or medications may impede recall; and if newborns are separated from their mothers to deliver care or interventions [21–23, 25, 26] (Additional file 2).

Institutional birth rates are increasing, with over three-quarters of births worldwide now in facilities [27], and many countries are starting to include newborn data within their routine systems [28–30] in line with multiple global initiatives [31–33]. Hence, routine facility data collected through health management information systems (HMIS) have potential as a source for coverage, yet validation research has focussed on survey-reported data. To our knowledge, no studies have assessed register-recorded coverage of breastfeeding, although some have assessed in-patient records and found low percent agreement between women’s recall and clinical records [34].

The timing and sequencing of INC practices represents one dimension of quality of care not generally included in large-scale survey tools [35], but that might have potential within routine HMIS. Skin-to-skin, immediate drying, delayed cord clamping (1–3 min after birth), and EIBF are all time bound interventions recommended soon after birth [5]. This research offers a unique opportunity to examine time-stamped data and assess to what extent we can accurately capture timing for these selected INC practices, and if these data could be useful to inform improvements in quality of care.

The *Every Newborn* Action Plan, endorsed by all United Nations member states, includes an ambitious measurement improvement roadmap [36, 37] underlining the imperative to validate indicators for maternal and newborn care. Measurement regarding care at birth needs to advance from health service contact alone (e.g., skilled attendance) to also tracking effective coverage, including content and quality of care [37, 38]. Accurate and more frequent data are essential to accelerate progress to Sustainable Development Goals, including Universal Health Coverage. However, many countries do not have regular and reliable data regarding INC practices. The EIBF indicator was prioritised within the *Every Newborn* measurement improvement roadmap [36, 37], given evidence of impact and survey data availability in many countries. This indicator was also proposed by WHO as a potential tracer for other INC indicators having plausibility of linkage; for example, EIBF may coincide with skin-to-skin care [39].

The *Every Newborn*-Birth Indicators Tracking in Hospitals (EN-BIRTH) study was an observational study of > 23,000 hospital births in three countries (Tanzania, Bangladesh, and Nepal); detailed methods and selected validity results are reported elsewhere [40, 41].

## Objectives

This paper is part of a supplement based on the EN-BIRTH multi-country validation study, ‘*Informing Measurement of Coverage and Quality of Maternal and Newborn Care’*. Here we focus on the measurement of EIBF and if EIBF can be used as a tracer for selected INC practices. There are four objectives:

1. **Assess NUMERATOR accuracy/validity** for measurement of EIBF in exit-interview survey of women’s report and in routine labour ward registers compared to clinical observation (gold standard). The **denominator** for EIBF is ‘live births’. This is consistent with current guidelines and measurement platforms, which also use live births [31, 42, 43].
2. **Review early initiation of breastfeeding as a potential TRACER indicator for other INC practices**: Compare observer-assessed coverage of EIBF to observer-assessed coverage of other immediate newborn care practices (skin-to-skin, drying, delayed cord clamping).
3. **Assess TIMING as a dimension of quality of care** by describing time to initiation of breastfeeding and the time to the selected INC practices using Kaplan Myer analysis shown by mode of birth.
4. **Evaluate BARRIERS AND ENABLERS** to routine labour ward register-recording through qualitative data collection regarding register design, and filling.

## Methods

EN-BIRTH included five comprehensive emergency obstetric and neonatal care (CEmONC) hospitals: Maternal and Child Health Training Institute, Azimpur, and Kushtia General Hospital in Bangladesh (BD); Pokhara Academy Health Sciences in Nepal (NP); and Muhimbili National Hospital and Temeke Regional Hospital in Tanzania (TZ) (Additional file 3). Data collection was from July 2017 to July 2018 (Additional file 4). Consenting women and newborns admitted to the labour and delivery wards were observed during birth and the immediate postpartum period. Observations were terminated once women and newborns were transferred out of labour and delivery ward. Exit interview surveys were conducted with women in the hospitals immediately after discharge (Additional file 4). All EN-BIRTH data collection tools are open source [44]. In line with current WHO recommendations, we defined EIBF as occurring within the first hour of life (Additional file 5) [45, 46]. For objectives 1 and 2, we excluded observations which lasted for less than 1 h after birth as inclusion of these observations could have caused an underestimate in EIBF coverage when compared with register-recorded or survey-reported data. Newborns would not have been counted irrespective of who initiated breastfeeding after the observation was terminated, but during their first hour of life.

**Fig. 1:**
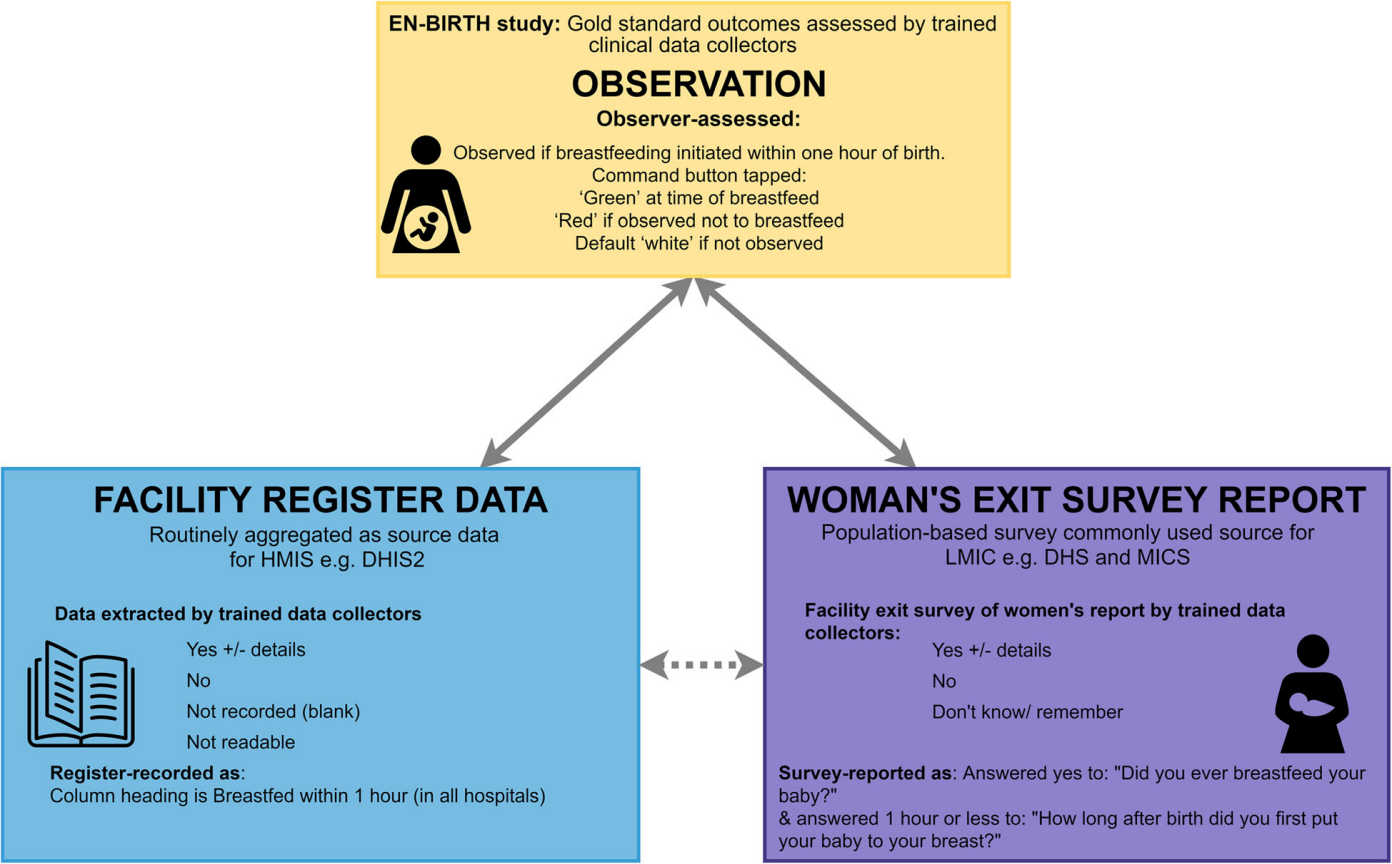
Immediate newborn care and breastfeeding practices validation design, EN-BIRTH study. *EN-BIRTH validation design comparing observation gold standard with register-recorded and women’s report on exit survey*

Gold standard observer-assessed coverage data were collected by trained clinical researchers using a custom-built android tablet-based application across the 24-h day. The software enabled observers to capture the practice whenever it occurred, and each entry was time-stamped (Fig. 1) [41]. Data collectors were trained to touch a specific button for recording the observed practice (skin-to-skin, drying, cord clamping or breastfeeding) once when it was initiated (colour coding the variable green on the application) (Additional file 5). Training materials were standardised across sites and supported with a printed manual available at each site [41]. In order to assess for bias, background characteristics of women observed for less than 1 h were compared with those of included cases.

One year of pre-study register data were extracted and compared to register-records during the study period to assess if the presence of external researchers in the hospital affected register recording [47]. Inter-rater reliability testing was completed for a subset of 5% of observed cases and data extraction [40]. All quantitative analyses were undertaken using Stata (version 14). Detailed information regarding the research protocol, methods, and overall validation analysis has been published separately [48].

Results are reported in accordance with STROBE statement checklists for cross-sectional studies (Additional file 6). We were granted ethical approval by institutional review boards in all implementing countries in addition to the London School of Hygiene & Tropical (Additional file 7).

## Labour ward registers

Pre-printed labour ward registers varied in design. During the study, the Bangladesh sites transitioned to a standardised national register (Additional file 3). Tanzania and the revised Bangladesh registers used for this analysis had a specific column for EIBF, both register designs used the wording “breastfed within 1 h of birth”. The Tanzania register requires staff to enter “yes” or “no” (Additional file 8), whilst the Bangladesh register required a tick for breastfed, and blank for not done. Nepal had no column to register-record breastfeeding. An overview of register design is available in Additional file 8.

### Objective 1: Numerator validation

Results were reported by hospital and mode of birth (vaginal and caesarean births). Random effects pooled estimates were used to calculate breastfeeding coverage across five hospital sites. We calculated percent agreement between observer-assessed coverage and measured coverage (survey or register), and the proportion of ‘don’t know’ responses from surveys, and ‘not recorded/not readable’ results from routine registers. We calculated individual-level validity metrics (sensitivity and specificity) for practices with ≥10 counts in 2 × 2 table columns. 95% confidence intervals (CIs) were calculated, assuming binominal distribution. Pokhara NP did not have a register column for breastfeeding and was therefore excluded from register-recorded analysis.

### Objective 2: Review early initiation of breastfeeding as a tracer indicator for other INC practices

Tracer coverage indicators reduce the number of indicators being tracked, but to be useful must accurately represent all other coverage indicators they replace. We aimed to assess if EIBF can be used as a tracer for other INC practices (skin-to-skin, drying, and delayed cord clamping). To this end, we calculated the percent agreement between pairs of observed interventions (EIBF and skin-to-skin, EIBF and drying, EIBF and delayed cord clamping), by summing the number of newborns who received both interventions and the number who received neither intervention, divided by the number of newborns observed.

### Objective 3: Assess timing as a dimension of quality of care

Quality of care is characterised across multiple domains of care provision. In this study, we assessed the timing of INC practices using the custom-built EN-BIRTH software and collected time-stamped observational data. Time to event analyses for skin-to-skin, drying, cord care, and breastfeeding initiation were undertaken using the Kaplan Meier method. All live births were included, excluding babies given bag and mask ventilation, or who weighed less than 1500 g. For this objective, results were censored when the observation terminated, or up to a maximum duration of 12 h of observation.

### Objective 4: Barriers and enablers to data collection

As part of the wider EN-BIRTH study, focus group discussions and in-depth qualitative interviews were conducted to understand the barriers and enablers to the use of routine registers in recording various aspects of perinatal care and outcomes [48]. Detailed qualitative methods and overall results are available in an associated paper [48]. In summary, we purposively sampled two groups of respondents: hospital health workers providing perinatal care in EN-BIRTH sites (nurses/midwives/doctors) and data collectors involved in the EN-BIRTH study (clinical observers/data extractors/supervisors) for participation in focus group discussions (FGD) and in depth interviews (IDI) (Additional file 9). Semi-structured IDI guides and semi-structured focus group guides were developed based on the Performance of Routine Information System Management (PRISM) conceptual framework [49]. Audio recordings of each interview were transcribed, translated, and managed with pre-identified codebook nodes into NVivo (version 12). Codes included constructs for technical, organisational, and behavioural factors. We also asked the participants to complete a checklist to assess which health worker usually provides care for breastfeeding, for documentation, and the order and timing of recording breastfeeding events in the register. These close-ended questions were asked by the researcher to respondents, immediately after their IDI (but not to FGD respondents).

## Results

**Fig. 2:**
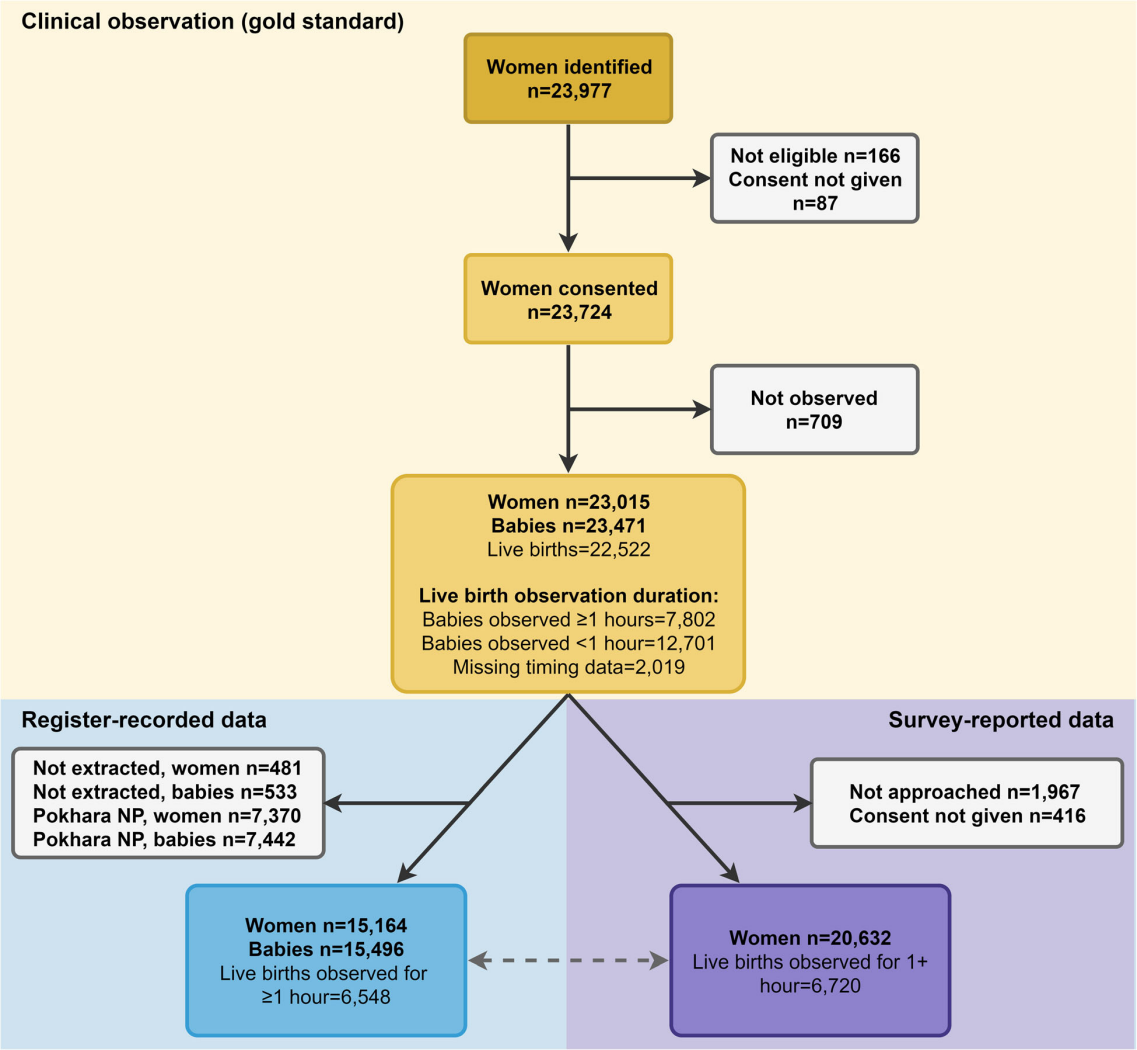
Flow diagram for immediate newborn care dataset, EN-BIRTH study (*n* = 23,015). *N* = 23,015 observed women. NP = Nepal. Pokhara (NP) had no register column for early initiation of breastfeeding; therefore Nepal is excluded from register-recorded data

This multi-country analysis included 23,724 consenting women, with 23,471 babies and 23,015 women being observed (Fig. 2). Overall, there were 22,522 live births. Observation data for at least 1 h was available for 7802 live newborns (single and multiple births), and there were 7412 newborn register-records, and 6720 exit-survey interviews. Table 1 presents the background characteristics of 7636 women and 7802 newborns observed for ≥1 h. More than two-thirds of births across all five sites were to women under the age of 30 years. Nearly 22% of women had a caesarean, although mode of birth varied widely across facilities. In Azimpur BD, Kushtia BD and Muhimbili TZ caesarean rates were highest at 53.3%, 30.9%, and 47.5%, respectively. Almost three quarters (77.3%) of births were full term (37+ weeks).

**Table 1:** Characteristics of women observed in labour and delivery wards, EN-BIRTH study (*n* = 7636)

	Bangladesh		Nepal	Tanzania		Total
	Azimpur	Kushtia	Pokhara	Temeke	Muhimbili	
	Tertiary	District	Regional	Regional	National	
	*n*(%)	*n*(%)	*n*(%)	*n*(%)	*n*(%)	*n*(%)
Total women	545	608	938	3771	1774	7636
**Woman’s Age**						
< 18 years	5(0.9)	1(0.2)	38(4.1)	10(0.3)	2(0.1)	56(0.7)
18–19 years	96(17.6)	46(7.6)	124(13.2)	429(11.4)	83(4.7)	778(10.2)
20–24 years	217(39.8)	257(42.3)	394(42)	1299(34.4)	345(19.4)	2512(32.9)
25–29 years	142(26.1)	164(27)	247(26.3)	943(25)	566(31.9)	2062(27)
30–34 years	66(12.1)	102(16.8)	112(11.9)	654(17.3)	478(26.9)	1412(18.5)
35+ years	19(3.5)	38(6.3)	23(2.5)	436(11.6)	300(16.9)	816(10.7)
**Woman’s education**						
No Education	7(1.3)	22(3.6)	25(2.7)	117(3.1)	32(1.8)	203(2.7)
Primary incomplete	24(4.4)	26(4.3)	31(3.3)	47(1.2)	16(0.9)	144(1.9)
Primary complete	78(14.3)	81(13.3)	47(5)	17(0.5)	2(0.1)	225(2.9)
Secondary incomplete	181(33.2)	237(39)	196(20.9)	2281(60.5)	617(34.8)	3512(46)
Secondary complete	229(42)	236(38.8)	608(64.8)	1292(34.3)	1097(61.8)	3462(45.3)
Don’t know	26(4.8)	6(1)	31(3.3)	17(0.5)	10(0.6)	90(1.2)
**Gestational age at admission (weeks)**						
< 28 weeks	1(0.2)	3(0.5)	0(0)	1(0)	8(0.5)	13(0.2)
28–31 weeks	0(0)	11(1.8)	0(0)	26(0.7)	89(5)	126(1.7)
32–36 weeks	110(20.2)	123(20.2)	47(5)	843(22.4)	469(26.4)	1592(20.8)
37+ weeks	434(79.6)	471(77.5)	891(95)	2901(76.9)	1208(68.1)	5905(77.3)
**Mode of birth**						
Vaginal birth	255(46.8)	420(69.1)	799(85.2)	3581(95)	931(52.5)	5986(78.4)
Caesarean section	290(53.2)	188(30.9)	139(14.8)	188(5)	842(47.5)	1647(21.6)
Missing	0(0)	0(0)	0(0)	2(0.1)	1(0.1)	3(0)

*N* = 7636 women and 7802 newborns observed for at least 1 h

### Objective 1: Numerator validation

**Fig. 3:**
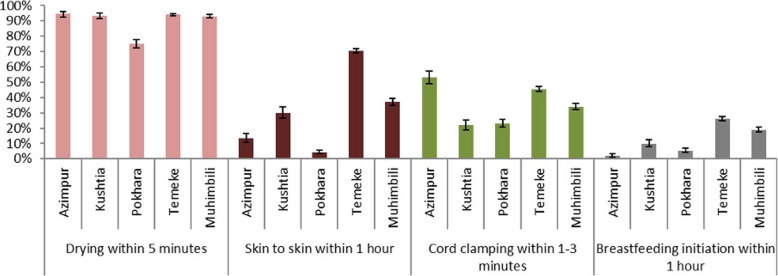
Observer-assessed coverage of immediate newborn care practices, EN-BIRTH study. Drying (*n* = 7784); skin-to-skin (*n* = 7773); Cord clamping within 1–3 min (*n* = 7791); breastfeeding initiation within 1 h (*n* = 7802). Timing parameters as recommended by the World Health Organisation, WHO recommendations on newborn health: guidelines approved by the WHO Guidelines Review Committee. 2017, Geneva

Coverage of EIBF was 10.9% (95% CI 3.8–21.0) for births observed ≥1 h (Fig. 3). Coverage was highest in Temeke TZ at 26.0% and lowest in Azimpur BD at 1.8%, where the caesarean section rate was 53.2% (Fig. 3). For caesarean births overall, the EIBF rate was 2.4% (95% CI 1.2–3.9) compared to 14.4% (95% CI 5.4–26.7) for vaginal births (Additional file 10).

**Fig. 4:**
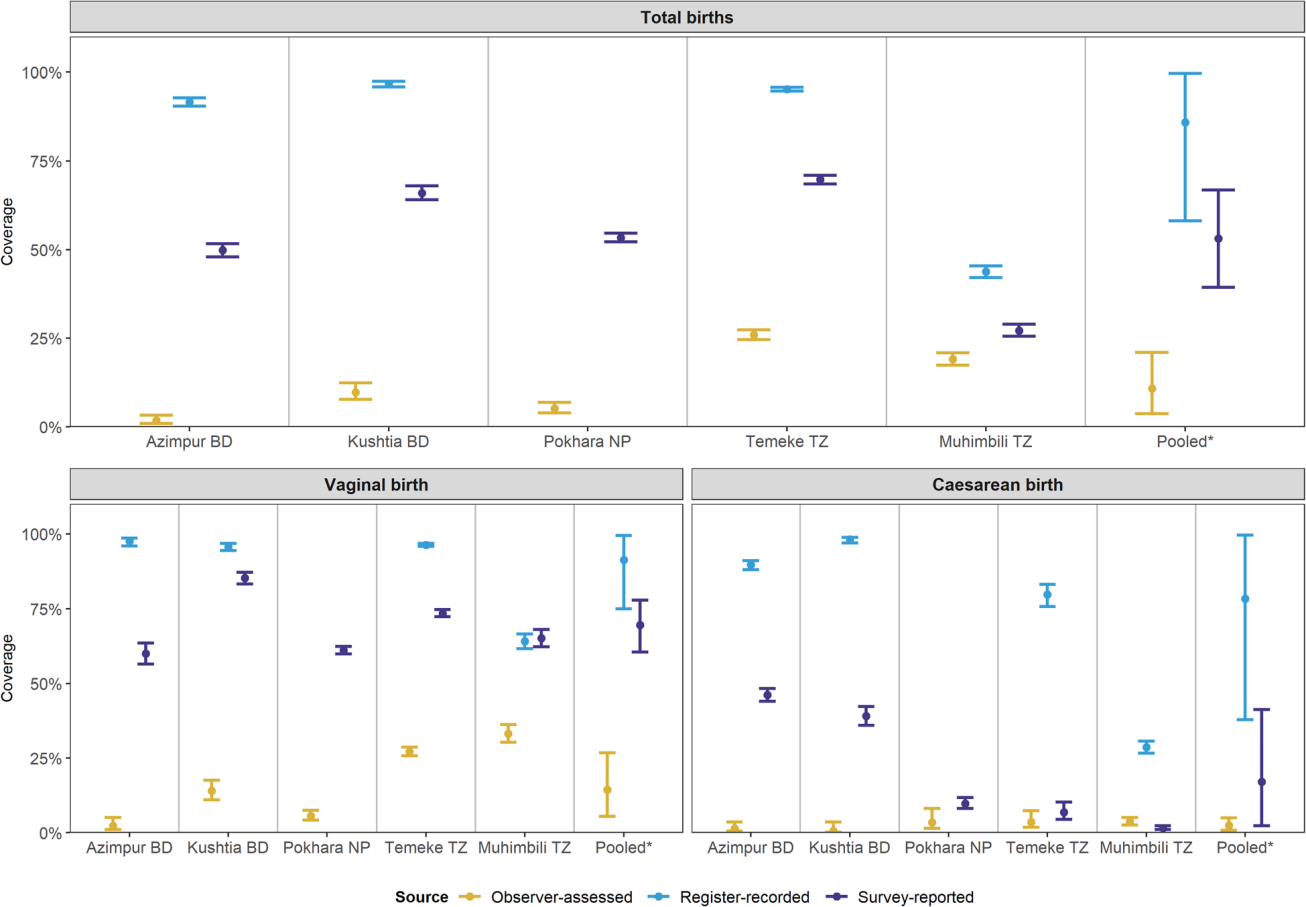
Coverage rates for early initiation of breastfeeding measured by observation, register and exit-survey, EN-BIRTH study (*n* = 7802). *N* = 7802 babies observed ≥1 h of birth. Bangladesh (BD); Nepal (NP); Tanzania (TZ). Pokhara (NP) had no register column for breastfeeding [41]

Register-recorded coverage was over-estimated in all sites with a column for this data element (Fig. 4, Additional file 8). Survey-reported coverage of “put to breast” was also higher than the observed prevalence. Percentage agreement for register-recorded data was 24.6% (95% CI 8.5–45.7) with high sensitivity 93.2% (95%CI 68.7–100) and low specificity 13% (95%CI 0.0–43.5) (Additional file 11). By facility, Kushtia BD (98.2%) and Temeke TZ (97.3%) had the highest sensitivity, while specificity ranged from 2.8% (95%CI 1.6–4.7) in Kushtia BD to 55.4% (95%CI 52.8–58.0) in Muhimbili TZ (Additional file 11). Sensitivity was 93.8% (95% CI 70.7–100.0) for vaginal births and 27.6% (95% CI 12.7–47.2) for caesarean births. Specificity of register-recorded coverage was 8.9% (95% CI 0.2–27.5) for vaginal births and 69.4% (95% CI 66.1–72.5) for caesareans (Additional file 11).

Percentage agreement for the survey-report was 53.8% (95% CI 40.2–67.2) with a sensitivity of 76.9% (95% CI 70.7–82.7), and specificity of 50.0% (95% CI 32.3–67.7). Sensitivity was 82.5% (95% CI 76.4–88) for vaginal births and 0.0% (95% CI 0.0–2.6) for caesarean births. The percentage agreement was highest in Temeke TZ (74.8%) and lowest in Kushtia BD (41.9%). Specificity of survey-report was 35.9% (95% CI 25.8–46.7) for vaginal births and 85.3% (95% CI 62.6–98.5) for caesareans (Additional file 10). Background characteristics for participants with ≥1 h of observation and those observed for less than 1 h were assessed and showed that a larger proportion of women observed for less than 1 h had a caesarean birth (Additional file 12).

### Objective 2: Assess agreement between EIBF with other INC practices

We assessed coverage of four INC practices: skin-to-skin contact, drying, delayed cord clamping, and EIBF using observation data (Fig. 3). Drying within 5 min after birth was over 90% in all hospitals apart from Pokhara (75.0%). Provision of skin-to-skin contact within 1 h of birth ranged from 13.5% of babies (Azimpur BD) to 70.5% (Temeke TZ). Cord clamping was universal, but timing varied between facilities with less than half of babies receiving delayed cord clamping during the optimum 1–3 min window.

**Fig. 5:**
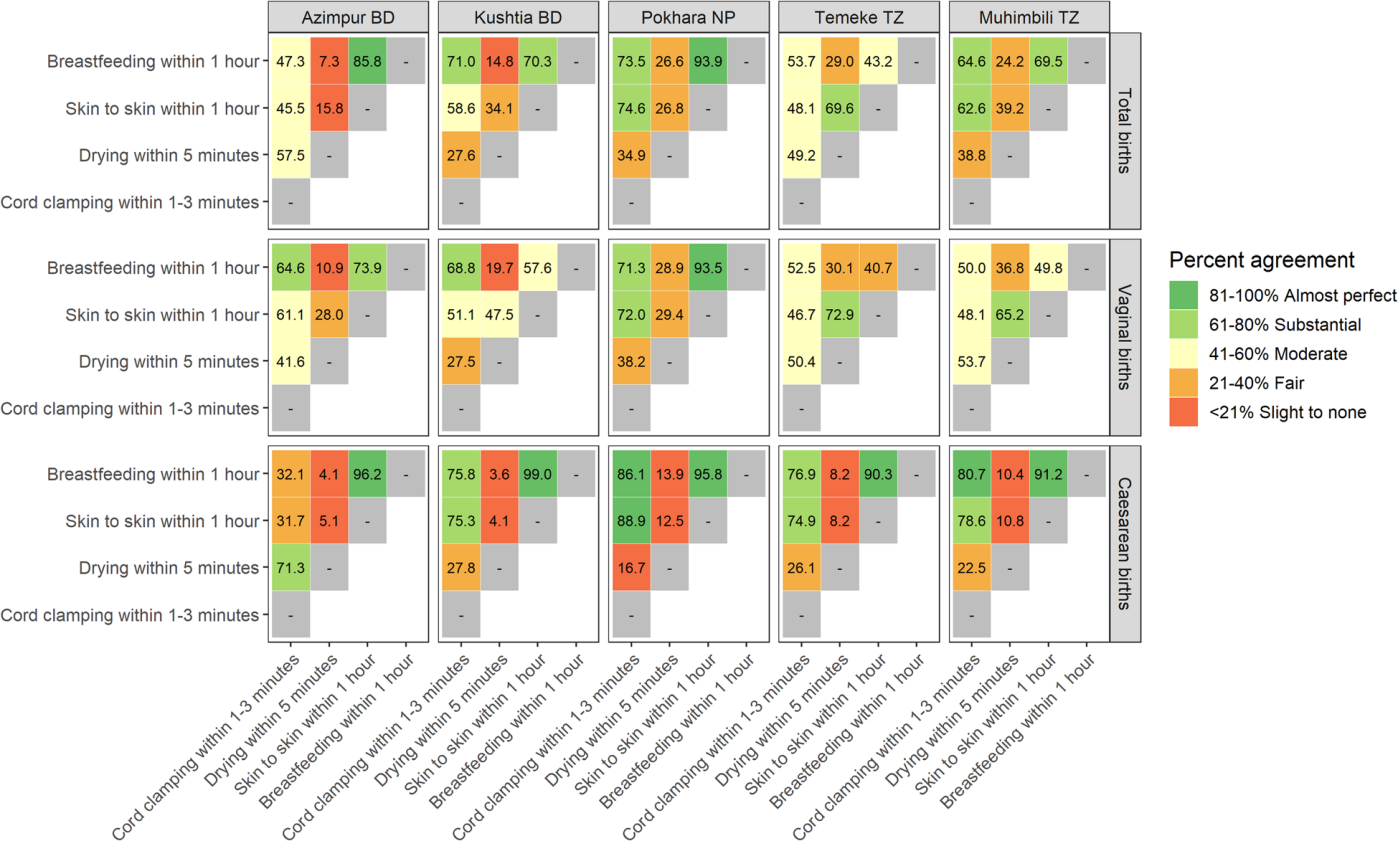
Agreement between observer-assessed immediate newborn care practices, EN-BIRTH study (*n* = 7802). *N = 7802 babies observed ≥ 1 h of birth. Bangladesh (BD); Nepal (NP); Tanzania (TZ). Observation data from Azimpur and Pokhara excluded due to poor inter-rater reliability for observation*

Observed coverage of EIBF was low in all facilities; consequently, it was not possible to assess the breastfeeding relationship with high coverage INC practices. The exception is skin-to-skin contact during the first hour, which demonstrated close percent agreement in four facilities: 93.9% in Pokhara NP, 85.8% in Azimpur BD, 70.3% in Kushtia BD and 69.5% in Muhimbili TZ. Using Kappa cut-offs, delayed cord clamping had a moderate-to-good agreement with EIBF, ranging from 47.3% in Azimpur BD to 73.5% in Pokhara NP. Percent agreement between EIBF and drying was poor and ranged from 7.3% in Azimpur BD to 29.0% in Temeke TZ (Fig. 5).

### Objective 3: Assess timing as a marker of quality of care

**Fig. 6:**
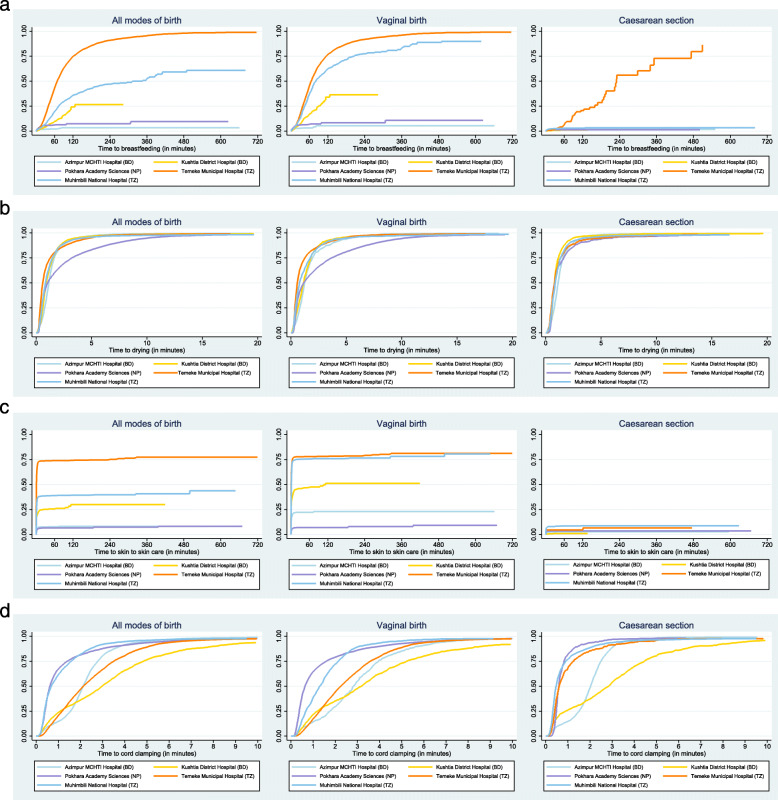
Kaplan-Meier plots of timing for immediate newborn care practices, EN-BIRTH study. *a. Breastfeeding initiation (All: 16,511, Vaginal births: 11,564, Caesarean births: 4944). b. Initiation of drying (All; 18,585, Vaginal births: 12,774, Caesarean births: 5808). c. Skin-to-Skin initiation (All: 17218, Vaginal births:12,199, Caesarean births: 5016). d. Cord-clamping (All: 18,586, Vaginal births: 12,775, Caesarean births: 5808). * Bangladesh (BD); Nepal (NP); Tanzania (TZ).

Kaplan Meier curves were plotted, showing the time from birth to initiation of skin-to-skin, drying, cord clamping, and breastfeeding (Fig. 6). Temeke TZ had the maximum probability of EIBF with a median time to initiation very close to 1 h. This was followed by Muhimbili TZ, however the median time was nearly 3 h. For vaginal births, the results were similar to the overall estimations. The probability of EIBF in Kushtia, Pokhara, and Azimpur within 1 h was lower than 0.3. For caesarean births EBFI was well after 1 h in all facilities with a median time of 240 min in Temeke TZ, the best performing facility.

The timing of drying was consistent across all five facilities and all modes of birth, with almost all babies dried within 5 min. Median time for drying was around 1 min in four facilities but slower in Pokhara NP (Fig. 6). In Temeke TZ and Muhimbili TZ, the median time was close to 1 min for initiation of skin-to-skin for vaginal births compared to 1 h in Kushtia BD. Babies born in Azimpur BD and Pokhara NP were least likely to get skin-to-skin contact in the first hour of life. The probability of skin-to-skin initiation for caesarean births was less than 0.1 in the first hour (Fig. 6). For vaginal births, the median time for cord clamping was between 1 and 3 min in Azimpur BD, Temeke TZ and Muhimbili TZ. Babies born in Pokhara NP were likely to have cord clamped before 1 min, while this was over 3 min in Kushtia BD (Fig. 6). For caesarean births, median time for cord clamping was less than 1 min except for in Azimpur BD and Kushtia BD.

### Objective 4: Barriers and enablers to data collection

**Fig. 7:**
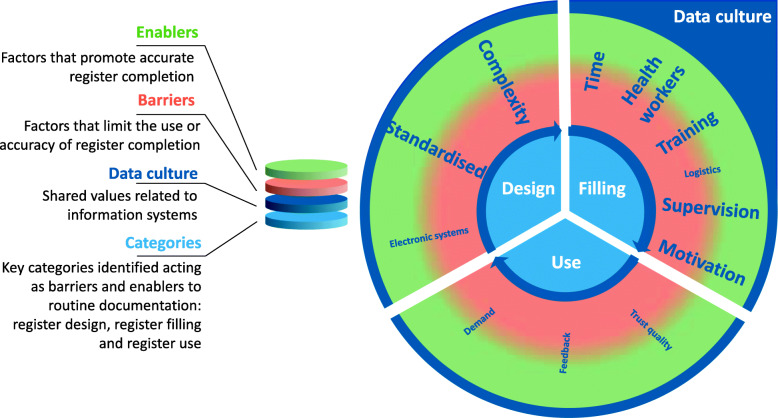
Barriers and enablers to routine register recording for immediate newborn care practices, EN-BIRTH study. This figure illustrates the overall barriers and enablers to facility-based data collection identified by EN-BIRTH participants. The **bold text** are the issues specific to immediate newborn care. The transition from red to green is a reminder that most factors identified by participants could serve as either a barrier or enabling factor depending on the facility-level resources and management

Three main categories were identified as influencing data collection and use in the EN-BIRTH study overall qualitative analysis: 1) register design, 2) register filling and 3) register use [48]. Register design and filling were influenced by the complexity of local data collection systems and time pressures faced by frontline staff. Figure 7 shows a summary of barriers and enablers for recording of breastfeeding practices as identified in the EN-BIRTH study. No respondents cited use of register data regarding breastfeeding.

### Register design

Both health workers and EN-BIRTH study clinical observers reported factors related to register design, notably the complexity of the documentation system, as a major barrier to recording in registers. One site had no column at all for EIBF, while staff in other hospitals reported duplicitous data demands with the same data elements being recorded in multiple documents:

*“There are many registers, it takes time to do all the documentation.”*

-Health worker, Muhimbili TZ

In Muhimbili TZ, EIBF was documented in a national labour ward register before being tallied by hand and input into the HMIS. Breastfeeding initiation was also supposed to be recorded on the woman’s file, case notes, treatment sheet, and in the “informal midwifery book”.

### Register filling

Respondents stated barriers to register filling included valuing completeness over accuracy. Data collectors in Tanzania reported that EIBF may be recorded in the register before newborns had even started breastfeeding:

*“ … the nurse usually writes that the baby has been breastfed, even if by that time the baby might not have been breastfed.”*

-Data collector, Temeke TZ

These findings were consistent with evidence from Bangladesh data collectors, and are reflected in the low observed breastfeeding coverage compared with high register-recorded practice in both sites. Multiple locations for documentation contributed to the complexity of the record-keeping system and these challenges were compounded when breastfeeding was initiated after discharge from the labour wards:

*“We don’t fill information about first time breast-feeding because they start it in other places [wards].”*

-Health worker, Muhimbili TZ

Respondents in all five sites also reported that breastfeeding was not routinely initiated or recorded in the operation theatres, this was especially the case for Bangladesh:

*“Breastfeeding is not done in the operation theatre. They never do it in operation theatres.”*

-Data collector, Kushtia BD

*“They usually do not initiate it in the in the theatre, it is initiated in the post-caesarean ward.”*

-Data collector, Temeke TZ

Across all sites, the primary midwifery or nursing carer was responsible for documentation for women having vaginal births, except Pokhara NP where labour ward registers do not include a column for breastfeeding initiation (Additional file 13). Respondents did not know who would record breastfeeding if it was actually done after caesarean section in the operating theatre (Additional files 13 and 14).

Data collectors and health workers reported that breastfeeding in Bangladesh is usually assisted by nurses or women’s attendants and is documented in the neonatal register, case notes, discharge letter, and monthly summary sheet. In Nepal, nurse-midwives advise women to initiate breastfeeding within 1 h, but there is no register-recorded documentation.

*“We advise the patient, we say, to feed milk within one hour. We have written in the chart to encourage breastfeeding, but it’s not there in registers.”*

-Health worker, Pokhara NP

Health workers in all three settings reported being busy, and that data recording could be time consuming:

*“* … *documentation requires time. In the ward we have 35-40 patients, we need to discharge, fill registers, make birth certificates so time is required.”* -Health worker, Pokhara NP

There was a potential conflict between administrative responsibilities, such as recording and reporting of data, and provision of clinical care:

“*You have to … respond to her with whatever she wants and [you] forget to document*”

-Health worker, Muhimbili TZ

## Discussion

Breastfeeding indicators are rightfully part of the WHO core 100 global indicators for child health and nutrition, given breastfeeding has strong evidence of high impact for reducing mortality and morbidity [5–8, 16, 18, 50, 51]. It has been measured in large-scale, population-based household surveys for decades (Additional file 1). Importantly, breastfeeding is also considered to be a marker of respectful maternity care and baby friendly services promoting zero separation of women and their newborns. EN-BIRTH’s large sample size and time-stamped data allowed us to assess validity of measures in both surveys and registers, examine the relationship of EIBF with other immediate newborn care practices, and also to consider differences between vaginal and caesarean births. Coverage of initiation of breastfeeding within 1 h was shockingly low (10.9, 95% CI 3.8–21.0 overall) and very few babies born by caesarean were breastfed, even within several hours. Our results show that EIBF was over-estimated in both register-recorded and survey-reported data compared to the gold standard of observation.

EIBF was harder to measure than most of the other indicators assessed for EN-BIRTH and has also been found to have low accuracy in other survey validation studies [51] (Additional file 2). Over-estimation of EIBF in both survey and registry data could be due to three possible reasons. Firstly, inaccuracies in reporting timing, whereby the newborn was breastfed, but after 1 h. There are well recognized issues for accurate report of timing, and evidence suggests these issues are exacerbated around the time of birth and the immediate postnatal period when both women and health workers may misjudge time [22, 25]. In addition, recent evidence from eight countries in Asia and the Pacific suggests a strong dose relationship between skin-to-skin and initiation of breastfeeding within 90 min following birth [18]. These findings suggest that the window of breastfeeding initiation may be wider than 1 h, and highlight the importance of ensuring health workers have adequate training and support in the implementation of early breastfeeding counselling.

Secondly, breastfeeding is a multistep process and it is possible that data collectors, health workers, or women may identify different parts of the breastfeeding process as the time of EIBF; such as baby put to breast, baby latched, or baby sucking. We note that breastfeeding initiation is not a one-time, easily recorded event like cord cutting or uterotonic injection. EN-BIRTH data collectors received standardised training on observing “initiation of breastfeeding” (Fig. 1, Additional file 4), but may still have applied their own interpretation to the exact time of initiation. In the current DHS and MICS survey question structure, women are asked, “Did you ever breastfeed your baby?” and then, “How long after birth was the baby was put to breast?” which is equally open to interpretation, and counting different points in the process of initiation [52]. Formative research could help better understand how these processes are interpreted. For example, if register design can improve accuracy by including one part of the process of EIBF, such as “put to breast” or sucking.

Thirdly, breastfeeding may be misreported by health workers or by women, possibly deliberately affected by social desirability for approval [22, 25]. Qualitative results suggested that the documentation culture in Bangladesh and Tanzania valued register completeness over accuracy, which exposes the need for training and supportive supervision to improve the accuracy of information included in registers. Health workers were divided across many tasks and did not always prioritise supporting women in initiating breastfeeding, nor accurate documentation. These testimonies also highlight the heavy workload on health providers, with consequences for how staff prioritise and complete their tasks, and might increase pressure for staff to record what they believe is the desirable answer [53]. Local monitoring and supervision to track different quality of care dimensions for breastfeeding are needed in the study settings, alongside practical facility-level solutions such as designing the ward layout to ensure record keeping can be completed in a convenient location near service users and the clinical area, and implementation of local protocols and training programs. However, changing EIBF and documentation practices is likely to also require health system actions that encompass improvements to human resources, infrastructure, supply and mechanisms for accountability [54, 55].

Drying of the newborn and skin-to-skin contact were challenging to measure in survey report for the EN-BIRTH study [56], and this is consistent with other research [22, 24, 25, 34]. Indeed, accuracy is expected to worsen over the two to five-year timespan used for DHS and MICS, compared to the exit survey timing in EN-BIRTH. Skin-to-skin is currently included in the DHS core questionnaire, drying in the DHS optional newborn module, and delayed cord clamping is not included in DHS or MICS (Additional file 1). For drying, survey-reported percent agreement was > 80% in 4/5 hospitals, but for skin-to-skin initiation was < 50% in three hospitals [56]. Results regarding individual level validation for survey-report of these INC indicators are detailed in a companion paper [56]. Cord cutting and drying or clamping are universally practiced for most births; quality of care improvement requires data on timing, and hygienic practices which are better assessed via audit, and other facility-level clinical quality improvement approaches. As such, we do not recommend inclusion of questions in surveys regarding cord clamping, drying, or immediate skin-to-skin for all babies (which differs from kangaroo mother care) [57].

Our observation data suggests EIBF was a good tracer indicator for skin-to-skin initiation within 1 h of birth in four of five assessed facilities (Azimpur BD, Kushtia BD, Pokhara NP, and Muhimbili TZ). There is compelling plausibility for the agreement between skin-to-skin and breastfeeding [18]. We also found good agreement between EIBF and delayed cord clamping in three facilities (Kushtia BD, Pokhara NP, and Muhimbili TZ). Coverage of delayed cord clamping and immediate drying was very high while coverage of EIBF was very low; EIBF in this study was not related to immediate drying, although we note that drying was practiced rapidly for virtually all newborns and EIBF was very low. This echoes prior secondary analysis of DHS data, which reported EIBF to be poorly correlated to other INC practices, although we note that the correlated data were based on survey-report with low accuracy, and thus had inherent limitations [58].

Our time-to-event analysis using the Kaplan Meier curves highlights the rapid timing of skin-to-skin initiation drying, and cord clamping, but major delays in breastfeeding, especially for babies born via caesarean. Given the increasing rate of caesareans, this represents an urgent research gap [59]. One EN-BIRTH hospital had an observed caesarean rate > 70%, which is high – double the recommended acceptable range of 10–15% [59]. Given the importance of INC practices, and especially the relationship between EIBF and skin-to-skin [18], urgent work is required to better understand and address the barriers and enablers for newborn care after caesarean birth, in addition to reducing non-medically indicated caesarean sections.

In these CEmONC hospitals, low rates of breastfeeding indicate gaps in quality of care. Given the well-evidenced, extensive benefits of EIBF, low coverage and delays are startling and may reflect separation of mother and baby. Breastfeeding initiation is crucial for establishing breastfeeding and for multiple other benefits for mother and baby [5]; hence other essential newborn care interventions such as vitamin K, eye care, immunisations, and assessment of birthweight, gestational age, or congenital conditions should not be prioritised above uninterrupted skin-to-skin and EIBF where possible. More work to assess sequencing and prioritisation of practices is required.

Register design also plays a role, the Pokhara NP register did not have a column to capture EIBF. In three out of four EN-BIRTH sites with a specific column, register-recorded coverage was above 90%. In Tanzania, Temeke and Muhimbili had different register-recorded coverage (95.3% and 43.8% respectively) despite sharing the same register design and having similar observer-assessed EIBF rates (26% and 19.1% respectively). Hospitals in Bangladesh introduced revised registers during the study period, and register-recorded breastfeeding coverage in Azimpur increased from 0 to > 90%, and in Kushtia from 57.3 to 96.8%, despite a maximum observer-assessed EIBF coverage of 9.8% [40]. These findings suggest that a focus on data accuracy is important, rather than register completeness alone. Further research regarding register filling and context to understand better these variations in performance, which may be rooted in facility-specific differences such as governance and leadership, could help. Facilitating ownership and use of data could also support improved data quality [60], especially in the operating theatres where health workers reported being unclear on who was responsible for recording in registers, or what data were used for reporting in HMIS (Additional files 13 and 14). Introducing data quality assurance systems, training on indicator definitions, and receiving feedback on data could help improve recording practices [61].

### Strengths and limitations

Strengths of this study include the large sample size, and rigorous multi-country design with gold standard with direct observation by clinically trained observers. Observer data could be subject to errors, but this risk was minimised through a custom-built electronic data capture system, standardised training and refresher sessions, and quality assurance through double observation and data entry [41].

However, there were also limitations. Observation was discontinued when women were transferred out of labour and delivery wards, so we were unable to record EIBF beyond the immediate postpartum period. As the current definition of EIBF includes a 1 h time period, the 12,701 women who were not observed for > 1 h needed to be excluded from the validation analysis. This may have introduced bias as women observed for ≥1 h were more likely to have had a vaginal birth (Additional file 10). Having observation data across the full sample for a longer period would enable a more detailed analysis regarding timing, especially validation at 2 h post-birth [11]. Despite low prevalence of data categorised as “not readable”, inter-rater reliability findings suggested poor agreement between register data extractors in Kushtia BD and Muhimbili TZ (Additional file 15). This highlights the potential challenges of data extraction and a need for evidence-based register design and implementation processes to ensure data quality as it moves up the HMIS [40].

Further research is needed to improve reliable and consistent measurement of the EIBF indicator, as well as comparability between survey and routine register data. Research on register design, implementation, and flow into HMIS is key. Root cause analysis tools could be adapted to identify local solutions for improving quality of maternal and newborn care in health facilities, in line with WHO standards [62].

## Conclusions

In this large multi-site study, most INC practices evaluated had suboptimal coverage and challenges in measurement. EIBF had very low coverage (less than one in five), and even lower for women with caesarean births. Given the global epidemic of caesareans, more focus on supporting women and newborns with EIBF is crucial. Unless measurement accuracy is improved, EIBF coverage changes may be missed. Register-recorded and survey-reported coverage both over-estimated observed coverage of EIBF, demonstrating a need for further research to improve instructions and register design/survey questions. Our analysis suggests that agreement between EIBF and skin-to-skin initiation is high. However, immediate drying and delayed cord clamping are even more challenging to measure in surveys and unlikely to be captured in registers, so they will likely require special audits and studies. Renewed focus is needed to promote zero separation of women and their babies, increase coverage of EIBF and INC practices irrespective of mode of birth, and to ensure and measure INC practices including respectful care practices for every woman and their newborn at birth.

## Supplementary Information

**Additional File 1.** Definition of immediate newborn care indicators (EN-BIRTH, DHS & MICS questionnaires).

**Additional File 2.** Previous studies regarding validation for measures of immediate newborn care practices.

**Additional File 3.** National context and number of births in EN-BIRTH study hospital.

**Additional File 4.** Data collection dates by site, EN-BIRTH study.

**Additional File 5.** Observation, survey and register indicator definitions, EN-BIRTH study.

**Additional File 6.** STROBE Checklist.

**Additional File 7.** Ethical approval by institutional review boards, EN-BIRTH Study.

**Additional File 8. ** Hospital register design and completion approaches by site, EN-BIRTH study (*n* = 6548).

**Additional File 9.** Respondents for focus group discussion and in-depth interviews for EN-BIRTH Study.

**Additional File 10. ** Individual-level validation in exit-survey report of early initiation of breastfeeding, EN-BIRTH study (*n* = 7802).

**Additional File 11.** Individual-level validation of register recording for early initiation of breastfeeding, EN-BIRTH study (n = 7802).

**Additional File 12. ** Characteristics of women observed in labour and delivery wards for < 1 h, EN-BIRTH study (*n* = 12,554).

**Additional File 13.** Assessment of routine recording responsibilities for breastfeeding, EN-BIRTH study.

**Additional File 14.** Register recording order and prioritisation for breastfeeding, EN-BIRTH study.

**Additional File 15.** Inter-observer agreement for early initiation of breastfeeding using Kappa, EN-BIRTH study.

## Data Availability

The datasets generated during and/or analysed during the current study are available on LSHTM Data Compass repository, https://datacompass.lshtm.ac.uk/955/.

## Abbreviations

BD: Bangladesh
CEmONC: Comprehensive emergency obstetric and neonatal care
CIFF: Children’s Investment Fund Foundation
DHS: The Demographic and Health Survey Program.
EIBF: Early initiation of breastfeeding
EN-BIRTH: *Every Newborn*-Birth Indicators Research Tracking in Hospitals study
HMIS: Health Management Information Systems
icddr,b: International Centre for Diarrheal Disease Research, Bangladesh
IHI: Ifakara Health Institute Dar es Salaam Tanzania
INC: Immediate newborn care
LMIC: Low and Middle Income Countries
LSHTM: London School of Hygiene & Tropical Medicine
MICS: Multiple Indicator Cluster Survey
NP: Nepal
PRISM: Performance of Routine Information System Management
TZ: Tanzania
UNICEF: United Nations Children's Fund
WHO: World Health Organization
