# Supplementary material for: Immediate newborn care and breastfeeding: EN-BIRTH multi-country validation study
Source: BMC Pregnancy Childbirth. 2021 Mar 26;21(Suppl 1):237. doi: 10.1186/s12884-020-03421-w (PMC7995709; doi:10.1186/s12884-020-03421-w)
Supplement: Supplementary file 1 — Additional File 1. Definition of immediate newborn care indicators (EN-BIRTH, DHS & MICS questionnaires). [file 12884_2020_3421_MOESM1_ESM.pdf]

Every Newborn BIRTH multi-country validation study: informing measurement of coverage and quality of maternal and newborn care

Immediate newborn care and breastfeeding: EN-BIRTH multi-country validation study

Additional File 1: Definition of Immediate Newborn Care indicators (EN-BIRTH, DHS & MICS questionnaires)

**Indicator**	**Question wording**		
	**EN-BIRTH**	**DHS-7 / DHS-8 Woman's Questionnaire and supplemental modules**	**MICS (Questionnaire for individual women, MICS6)**
**Immediate newborn care:**			
Baby dried or wiped immediately after birth	Was your baby dried or wiped immediately after birth (within a few minutes)?	NB1 (Newborn care supplemental module). Was (NAME) wiped dry within a few minutes after birth?	MN25. Was (name) dried or wiped soon after birth?
Baby placed naked against mother's skin	Was your baby placed on your chest, naked against your skin? PROMPT WITH PICTURE IF NECESSARY	DHS-7: 434. Immediately after the birth, was (NAME) put on your chest?<br>If "yes": 434A. Was (NAME)'s bare skin touching your bare skin?<br><br>DHS-8: 438. After the birth, was (NAME) put on your chest?<br>If "yes" 439. Was (NAME)'s bare skin touching your bare skin?	MN23. Immediately after the birth, was (name) put directly on the bare skin of your chest?<br>If necessary, show the picture of skin-to-skin position.<br>If "yes": MN24. Before being placed on the bare skin of your chest, was the baby wrapped up?
Early initiation of breastfeeding (within 1 hour after birth)	Did you ever breastfeed your baby?<br>If "yes": How long after birth did you first put your baby to your breast?	464 (DHS-7)/480 (DHS-8). Did you ever breastfeed (NAME)?<br>If "yes": 466 (DHS-7)/483 (DHS-8). How long after birth did you first put (NAME) to the breast?	MN36. Did you ever breastfeed (name)?<br>IF "YES": **MN37**. HOW LONG AFTER BIRTH DID YOU FIRST PUT (***NAME***) TO THE BREAST?
Birthweight measured	Was your baby weighed at birth?	427 (DHS-7)/442 (DHS-8). Was (NAME) weighed at birth?	MN33. Was (name) weighed at birth?
Anything applied to cord	After the cord was cut, was anything applied to the stump of the cord at any time?	NB7 (Newborn care supplemental module). Was anything applied to the stump of the cord at any time?	MN30. After the cord was cut and until it fell off, was anything applied to the cord?
Chlorhexidine applied to cord	What was applied to the cord? (Show tube)? PROBE: Anything else?	NB8 (Newborn care supplemental module). What was applied? Anything else?<br>If "chlorhexidine" not reported: CH2. Was chlorhexidine applied to the stump at any time?	MN31. What was applied to the cord?<br>Probe: Anything else?
Feeding support- Cup feeding		-	-
Phototherapy given	Did your baby receive any phototherapy during the hospital stay? For example, was your baby yellow and put underneath lights?	-	-

DHS: Demographic Health Survey, USAID [1].MICS: Multiple Indicator Cluster Survey, UNICEF [2]. Further details available [3]

References

1. **The DHS program** [https://dhsprogram.com/]accessed 10.09.20
2. **Multiple Indicator Cluster Surveys (MICS) Questionnaires 5 & 6** [http://mics.unicef.org/tools] accessed 10.09.20
3. Shafiqul AB, AB. Rahman, EA. Peven, K. Tashsina, T. Zaman, SB. Rahman, QS. Hossain, AT. KC, A. Shamba, D. Boggs, D. Blencowe, H. Day, LT. Ruysen, H. EN-BIRTH Collaborator Group. Arifeen, S. Lawn, JE. : **Assessing the validity of 33 maternal & newborn indicators in a survey of women's report: EN-BIRTH multi-country study informing measurement of coverage and quality** *BMC Pregnancy Childbirth* [IN PRESS].
