# Supplementary material for: Immediate newborn care and breastfeeding: EN-BIRTH multi-country validation study
Source: BMC Pregnancy Childbirth. 2021 Mar 26;21(Suppl 1):237. doi: 10.1186/s12884-020-03421-w (PMC7995709; doi:10.1186/s12884-020-03421-w)
Supplement: Supplementary file 2 — Additional File 2. Previous studies regarding validation for measures of immediate newborn care practices. [file 12884_2020_3421_MOESM2_ESM.pdf]

Every Newborn BIRTH multi-country validation study: informing measurement of coverage and quality of maternal and newborn care

Immediate newborn care and breastfeeding: EN-BIRTH multi-country validation study

Additional File 2: Previous studies regarding validation for measures of Immediate Newborn Care practices

	Sample	Maternal Exit Survey+	% Don't Knows	Follow up Survey	% Don't Knows	Maternity Register
**Early Initiation of Breastfeeding[1]**						
Bhattacharya (2019), Nigeria	1775, 426	AUC & IF	<5%	Neither	<5%	Not in register
Blanc (2016), Kenya	551	AUC only	<5%			
Blanc (2016), Mexico	449	AUC only	<5%			
McCarthy (2016), Kenya	662	IF Only	<5%	AUC & IF		
Stanton (2013), Mozambique	487 observed, 304 interviewed	Neither				
Broughton (2013), Afghanistan	600					49.3% (in patient notes)
**Dried within 5 minutes**						
Bhattacharya (2019), Nigeria	1472	AUC & IF	<5%	Analysis criteria not met	<5%	Not in register
Blanc (2016), Kenya	594	Analysis criteria not met	8.3%			
Blanc (2016), Mexico	-	-	-	-	-	-
McCarthy (2016), Kenya	662	Analysis criteria not met	8.4%	Analysis criteria not met	13.3%	-
Stanton (2013), Mozambique	508	IF only				
Broughton (2013), Afghanistan	-	Neither	-	-	-	-
**Initiation of skin to skin within 1 hour**						
Bhattacharya (2019), Nigeria	1775	AUC & IF	<5%	Neither AUC or IF	<5%	Not in register
Blanc (2016), Kenya	602	Neither	-	-	-	-
Blanc (2016), Mexico	-	-	-	-	-	-
McCarthy (2016), Kenya	662	Neither	<5%	Neither AUC or IF	5.3%	
Stanton (2013), Mozambique	508	AUC & IF				
Broughton (2013), Afghanistan	-	-	-	-	-	-

+AUC defined as ≥0.6, IF 0.75-1.25.

[1]Register-recorded as composite indicator with ''keeping warm''

Bhattacharya (2019), Nigeria. Blanc (2016), Kenya. Blanc (2016), Mexico. McCarthy (2016), Kenya. Stanton (2013), Mozambique. Broughton (2013), Afghanistan [1-6].

References

1. Bhattacharya AA, Allen E, Umar N, Usman AU, Felix H, Audu A, Schellenberg JR, Marchant T: **Monitoring childbirth care in primary health facilities: a validity study in Gombe State, northeastern Nigeria**. *Journal of global health* 2019, **9**(2).
2. Blanc AK, Diaz C, McCarthy KJ, Berdichevsky K: **Measuring progress in maternal and newborn health care in Mexico: validating indicators of health system contact and quality of care**. *BMC pregnancy and childbirth* 2016, **16**(1):255.
3. Blanc AK, Warren C, McCarthy KJ, Kimani J, Ndwiga C, RamaRao S: **Assessing the validity of indicators of the quality of maternal and newborn health care in Kenya**. *Journal of global health* 2016, **6**(1).
4. Broughton EI, Ikram AN, Sahak I: **How accurate are medical record data in Afghanistan's maternal health facilities? An observational validity study**. *BMJ open* 2013, **3**(4):e002554.
5. McCarthy KJ, Blanc AK, Warren CE, Kimani J, Mdawida B, Ndwidga C: **Can surveys of women accurately track indicators of maternal and newborn care? A validity and reliability study in Kenya**. *Journal of global health* 2016, **6**(2).
6. Stanton CK, Rawlins B, Drake M, dos Anjos M, Cantor D, Chongo L, Chavane L, da Luz Vaz M, Ricca J: **Measuring coverage in MNCH: Testing the validity of women's self-report of key maternal and newborn health Interventions during the peripartum period in Mozambique**. *PLoS One* 2013, **8**(5):e60694.
