# Supplementary material for: Immediate newborn care and breastfeeding: EN-BIRTH multi-country validation study
Source: BMC Pregnancy Childbirth. 2021 Mar 26;21(Suppl 1):237. doi: 10.1186/s12884-020-03421-w (PMC7995709; doi:10.1186/s12884-020-03421-w)
Supplement: Supplementary file 3 — Additional File 3. National context and number of births in EN-BIRTH study hospital. [file 12884_2020_3421_MOESM3_ESM.pdf]

Every Newborn BIRTH multi-country validation study: informing measurement of coverage and quality of maternal and newborn care

Immediate newborn care and breastfeeding: EN-BIRTH multi-country validation study

Additional File 3: National context and number of births in EN-BIRTH study hospital

**Country Context**	**Bangladesh**		**Nepal**	**Tanzania**	
**National mortality rates at start of EN-BIRTH study (2016)**					
MMR/ 100,000 live births [1]	176		258	398	
NMR/ 1000 live births [2]	21		22	22	
SBR/ 1000 total births [3]	25		18	22	
% Institutional Births (2016)[4]	47.1		57.4	62.6	
**EN-BIRTH Study Hospitals**					
**Name**	Maternal and Child Health Training Institute (MCHTI), Azimpur, Dhaka	Kushtia District Hospital	Pokhara Academy of Health Sciences	Temeke Regional Hospital, Dar es Salaam	Muhimbili National Hospital, Dar es Salaam
**Hospital type**	Tertiary	District	Regional	Regional	National
**Total births annual 2017-18**	3,346	2,887	9,422	11,609	8,233

References:

1. Division. WHOUWBGUNP: **Trends in maternal mortality: 1990 to 2015.** In.; 2015.
2. UNICEF W, World Bank, UN-DESA Population Division, : **Levels and trends in child mortality 2015**. In. New York 2015.
3. Blencowe H, Cousens S, Jassir FB, Say L, Chou D, Mathers C, Hogan D, Shiekh S, Qureshi ZU, You D: **National, regional, and worldwide estimates of stillbirth rates in 2015, with trends from 2000: a systematic analysis**. *The Lancet Global Health* 2016, **4**(2):e98-e108.
4. World Health organisation: **Global Health Observatory data repository**. In.
