# Supplementary material for: Immediate newborn care and breastfeeding: EN-BIRTH multi-country validation study
Source: BMC Pregnancy Childbirth. 2021 Mar 26;21(Suppl 1):237. doi: 10.1186/s12884-020-03421-w (PMC7995709; doi:10.1186/s12884-020-03421-w)
Supplement: Supplementary file 4 — Additional File 4. Data collection dates by site, EN-BIRTH study. [file 12884_2020_3421_MOESM4_ESM.pdf]

# Immediate newborn care and breastfeeding: EN-BIRTH multi-country validation study

Additional File 4: Data collection dates by site, EN-BIRTH study

| EN-BIRTH study | Bangladesh Azimpur Tertiary | Bangladesh Kushtia District | Nepal Pokhara Regional | Tanzania Temeke Regional | Tanzania Muhimbili National | All sites |
|---|---|---|---|---|---|---|
| **Tablet data collection dates** | 17/8/17 to 30/4/18 | 11/7/17 to 30/5/18 | 17/7/17 to 31/7/18 | 3/7/17 to 30/5/18 | 3/7/17 to 28/2/18 | |
| Duration | 8 months | 10 months | 1 year | 10 months | 7 months | |
| Original register used: | 17/8/17 to 18/10/17 | 25/8/17 to 27/9/17 (due to short supply) | Not applicable | Not applicable | Not applicable | |
| Revised register used: | 19/10/17 to 30/4/18 | 11/7/17 to 24/8/17 and 28/9/17 to 30/5/18 | Not applicable | Not applicable | Not applicable | |
| **Register extraction comparison dates to assess biases** | | | | | | |
| **Pre-study** | 1/1/16 to 31/12/16 | 1/1/16 to 31/12/16 | 1/4/16 to 31/3/17 | 1/1/16 to 31/12/16 | 1/1/16 to 31/12/16 | |
| Duration | 12 months | 12 months | 12 months | 12 months | 12 months | |
| **During/after-study** | 17/8/17 to 17/8/18 | 11/7/17 to 11/7/18 | 17/7/17 to 17/7/18 | 3/7/17 to 3/7/18 | 3/7/17 to 3/7/18 | |
| Duration | 12 months | 12 months | 12 months | 12 months | 12 months | |
| **Time elapsed between delivery and exit survey interview/ days** | n (%) | n (%) | n (%) | n (%) | n (%) | n (%) |
| **Total** | 2844 | 2331 | 6922 | 5752 | 2783 | 20632 |
| 0-1 day | 725 (25.5) | 1345 (57.7) | 5854 (84.6) | 5433 (94.5) | 1009 (36.3) | 14366 (69.6) |
| 2-3 days | 511 (18) | 846 (36.3) | 833 (12) | 181 (3.1) | 1098 (39.5) | 3469 (16.8) |
| 4+ days | 1599 (56.2) | 127 (5.4) | 154 (2.2) | 43 (0.7) | 597 (21.5) | 2520 (12.2) |
| Missing | 9 (0.3) | 13 (0.6) | 81 (1.2) | 95 (1.7) | 79 (2.8) | 277 (1.3) |
| Mean | 3.1 | 1.3 | 0.6 | 0.7 | 3.2 | 1.4 |
| Median | 4.0 | 0.0 | 0.0 | 0.0 | 2.0 | 1.0 |

Sample size was calculated to observe at least 106 observations per intervention per country, based on estimated coverage of intervention during formative research [1].

**References:**

1. Day LT, Ruysen H, Gordeev VS, et al. "Every Newborn-BIRTH" protocol: observational study validating indicators for coverage and quality of maternal and newborn health care in Bangladesh, Nepal and Tanzania. *Journal of Global Health* 2019; **9**(1).
