# Supplementary material for: Immediate newborn care and breastfeeding: EN-BIRTH multi-country validation study
Source: BMC Pregnancy Childbirth. 2021 Mar 26;21(Suppl 1):237. doi: 10.1186/s12884-020-03421-w (PMC7995709; doi:10.1186/s12884-020-03421-w)
Supplement: Supplementary file 5 — Additional File 5. Observation, survey and register indicator definitions, EN-BIRTH study. [file 12884_2020_3421_MOESM5_ESM.pdf]

Every Newborn BIRTH multi-country validation study: informing measurement of coverage and quality of maternal and newborn care

Immediate newborn care and breastfeeding: EN-BIRTH multi-country validation study

Additional File 5: Observation, survey and register indicator definitions, EN-BIRTH study

	Observation	Exit-Survey	Labour Ward Register
**Numerator**	Number of newborns breastfed within 1 hour of birth	Number of newborns put to the breast within one hour of birth	Number of newborns breastfed within one hour of birth
**Denominator**	Number of live births observed ≥1 hour after birth	All live births observed ≥1 hour after birth	All live births observed ≥1 hour after birth
**Source**	World Health Organization. 2018 Global Reference List of 100 Core Health Indicators (plus health-related SDGs) Geneva: World Health Organization; 2018. Licence: CC BY-NC-SA 3.0 IGO.<br>**Indicator**: Percentage of newborns breastfed within 1 hour of birth. Numerator: Number of newborns breastfed within 1 hour of birth. Denominator: Number of live births in a specified time period.	Demographic and Health Surveys (DHS-7 / DHS-8), Woman's Questionnaire and supplemental modules *The DHS program*. 2020 14.01.20]; Available from: https://dhsprogram.com/<br>Based on: World Health Organisation, UNICEF: Indicators for assessing infant and young child feeding practices: Part 2 Measurement. In. Geneva: World Health Organization; 2010<br>**Indicator**: Percentage of newborns put to the breast within one hour of birth. Numerator: number of newborns put to the breast within one hour of birth Denominator: number of newborns for which a complete interview was available.	Labour and Delivery Ward Registers in Azimpur and Kushtia (Bangladesh), Pokhara (Nepal, and Temeke and Muhimbili (Tanzania) (additional file 7).
**Data Collection**	Observed if breastfeeding initiated within one hour of birth.<br>Command button tapped:<br>''Green'' at time of breastfeed<br>''Red'' if observed not to breastfeed<br>Default ''white'' if not observed.	Women Asked:<br>''Did you ever breastfeed your baby?''<br>If "yes":<br>''How long after birth did you first put your baby to your breast?'' (immediately after birth or within 1 hour)	Register Colum Heading/ instructions (Bangladesh and Tanzania):<br>Breastfed within 1 hour [of birth]<br>Bangladesh: tick if yes, blank if no<br>Tanzania: write yes if yes and no if not.<br>Nepal: no column in register
**Next Steps**	Breastfeeding is a multi-step process including several actions: baby put to breast, nipple in mouth, attaches, feeds etc. As such, the 2010 Guideline, *Indicators for assessing infant and young child feeding practices: Part 2 Measurement*, proposes an indicator of: percentage of newborns put to the breast within one hour of birth. Alignment of the indicator for use across different measurement platforms is needed.	The activity ''put to breast'' requires explicit instruction to define what this action includes: e.g. any occasion baby is put near the breast, or if some attempt at feeding was made i.e. nipple in mouth	Global guidance is needed to define and align a recommended indicator for use in health management information systems, including for labour ward and delivery ward registers.

Variable Matrix	Source and availability of data			
**Indicator**	**Registration dataset**	**Observation**	**Routine registers**	**Maternal Recall survey**
Background characteristics of women	√			√√
Mode of birth		√√	√	√
Delivery outcome		√√	√	√
**Immediate *newborn care practice***				
Initiation of breastfeeding		√√	√√+	√√
Skin-to-skin		√√		√
Cord-cutting		√√		√
Drying		√√		√
Time of care		√√		Partial

√√ used for analysis (+not in Pokhara)
√ data available at source
