# Supplementary material for: Immediate newborn care and breastfeeding: EN-BIRTH multi-country validation study
Source: BMC Pregnancy Childbirth. 2021 Mar 26;21(Suppl 1):237. doi: 10.1186/s12884-020-03421-w (PMC7995709; doi:10.1186/s12884-020-03421-w)
Supplement: Supplementary file 8 — Additional File 8. Hospital register design and completion approaches by site, EN-BIRTH study (n = 6548). [file 12884_2020_3421_MOESM8_ESM.pdf]

Every Newborn BIRTH multi-country validation study: informing measurement of coverage and quality of maternal and newborn care

Immediate newborn care and breastfeeding: EN-BIRTH multi-country validation study

Additional File 8: Hospital register design and completion approaches for breastfeeding by site, EN-BIRTH study (n=6,548)

		**Bangladesh**				**Nepal**	**Tanzania**	
		**Azimpur**		**Kushtia**		**Pokhara**	**Temeke**	**Muhimbili**
		**Tertiary**		**District**		**Regional**	**Regional**	**National**
**Early Initiation of Breastfeeding (observed ≥1 hour)**		**Original**	**Revised**	**Original**	**Revised**			
**Register design:** Column allotted data element		no column	specific column	Non-specific column	specific column	no column	specific 2 columns	specific 2 columns
Column 1 heading			Breastfed within 1 hour	Newborn	Breastfed within 1 hour		Breastfed within 1 hour	Breastfed within 1 hour
Column 1: data element completed if breastfed			Tick	Breastfed	Tick		Yes (in Swahili)	Yes (in Swahili)
Column 1: data element completed if not breastfed			Blank (clear instruction)	Blank	Blank (clear instruction)		No (in Swahili)	No (in Swahili)
Column 2 heading							Feeding Baby	Feeding Baby
Column 2: data element completed if breastfed							EBF (= breast milk)	EBF (= breast milk)
							RF (= other milk)	RF (= other milk)
Column 2: data element completed if not breastfed							dash or No (in Swahili)	dash or No (in Swahili)
**Completeness**	Data element recorded in register		Not possible	Not possible	Not possible		97.7%	76.6%
**External Consistency**								
Indicator:	Observed coverage %		1.8%	9.8%	9.8%		26.0%	19.1%
Indicator:	Measured coverage - register recorded %		91.7%	95.3%	96.8%		95.3%	43.8%
Measurement gap:	Register recorded and observed		89.9% over-estimate	85.5% over-estimate	87.0% over-estimate		69.3% over-estimate	24.7% over-estimate

Key

no column for data element	>20%	Poor
non-specific column for data element	16-20%	Moderate
specific column	11-15%	Good
	6-10%	Very Good
	0-5%	Excellent

Completeness calculations are “not possible” for Bangladesh register instructions state, blank is to mean intervention/practice is not done.
Cut-off ranges adapted from WHO Data Quality Review, Module 2 “Desk review of data quality” [1]
N= 6,548 register-recorded live births observed for ≥1 hour after birth

References

1. World Health Organisation: **Data quality review: a toolkit for facility data quality assessment. Module 2: Desk review of data quality**

In. Geneva; 2017.
