# Supplementary material for: Immediate newborn care and breastfeeding: EN-BIRTH multi-country validation study
Source: BMC Pregnancy Childbirth. 2021 Mar 26;21(Suppl 1):237. doi: 10.1186/s12884-020-03421-w (PMC7995709; doi:10.1186/s12884-020-03421-w)
Supplement: Supplementary file 10 — Additional File 10. Individual-level validation in exit-survey report of early initiation of breastfeeding, EN-BIRTH study (n = 7802). [file 12884_2020_3421_MOESM10_ESM.pdf]

Every Newborn BIRTH multi-country validation study: informing measurement of coverage and quality of maternal and newborn care

Immediate newborn care and breastfeeding: EN-BIRTH multi-country validation study

Additional File 10: Individual-level validation in exit-survey report of early initiation of breastfeeding, EN-BIRTH study (n=7,802)

	Bangladesh				Nepal		Tanzania				All sites						
	Azimpur Tertiary		Kushtia District		Pokhara Regional		Temeke Regional		Muhimbili National								
	%	95%CI	%	95%CI	%	95%CI	%	95%CI	%	95%CI	Pooled Random Effects		Q	df	p-val.	i²	τ²
**Survey reported - All modes of birth**																	
Observer coverage %	1.8	(1.0,3.3)	9.8	(7.7,12.4)	5.3	(4.0,6.9)	26.0	(24.6,27.4)	19.1	(17.3,20.9)	10.9	(3.8,21.0)	536.8	4	0.000	99.3%	0.101
Survey reported coverage %	49.8	(48.0,51.7)	66.0	(64.0,67.9)	53.4	(52.2,54.6)	69.7	(68.5,70.9)	27.2	(25.5,28.9)	53.2	(39.4,66.8)	1536.9	4	0.000	99.7%	0.101
"Don't know" responses %	0.0	(0.0,0.3)	0.2	(0.1,0.5)	0.3	(0.2,0.5)	0.8	(0.6,1.0)	2.5	(2.0,3.2)	0.6	(0.1,1.3)	125.2	4	0.000	96.8%	0.008
**Current survey - Count "don't know" as "no"**																	
Sensitivity % (95% CI)	50.0	(18.7,81.3)	86.9	(75.8,94.2)	63.8	(48.5,77.3)	77.8	(74.9,80.5)	77.5	(71.8,82.5)	76.9	(70.7,82.7)	11.4	4	0.023	64.8%	0.013
Specificity % (95% CI)	52.6	(48.2,56.9)	36.9	(32.8,41.1)	53.9	(50.4,57.3)	31.5	(29.7,33.4)	74.7	(72.0,77.2)	50.0	(32.3,67.7)	666.5	4	0.000	99.4%	0.169
Percent agreement (TP+TN/n) %	52.5	(48.3,56.7)	42.0	(38.1,45.9)	54.4	(51.1,57.7)	43.8	(42.1,45.5)	75.2	(72.9,77.4)	53.8	(40.2,67.2)	445.5	4	0.000	99.1%	0.097
Positive Predictive Value % (95% CI)	2.0	(0.6,4.6)	13.4	(10.2,17.2)	7.3	(5.0,10.2)	29.0	(27.2,30.9)	40.7	(36.2,45.2)							
Negative Predictive Value % (95% CI)	98.2	(95.9,99.4)	96.2	(92.6,98.3)	96.3	(94.2,97.8)	79.8	(77.1,82.3)	93.7	(91.9,95.2)							
Inflation Factor and "Bias"	25.0	large	6.5	large	8.7	large	2.7	large	1.9	large							
AUC and "accuracy"	0.5	low	0.6	moderate	0.6	low	0.6	low	0.8	high							
**Consider only "yes" and "no" (exclude don't know)**																	
Sensitivity (95% CI)	50.0	(18.7,81.3)	88.3	(77.4,95.2)	63.8	(48.5,77.3)	78.3	(75.4,81.0)	78.4	(72.8,83.3)	77.5	(70.9,83.5)	12.7	4	0.013	68.5%	0.015
Specificity (95% CI)	52.5	(48.1,56.8)	36.8	(32.7,41.0)	53.8	(50.3,57.2)	31.0	(29.2,32.9)	74.0	(71.3,76.6)	49.7	(32.1,67.3)	650.4	4	0.000	99.4%	0.167
Percent agreement (TP+TN/n) %	52.4	(48.2,56.7)	41.9	(38.0,45.9)	54.3	(51.0,57.6)	43.5	(41.8,45.2)	74.8	(72.4,77.1)	53.7	(40.2,66.9)	431.6	4	0.000	99.1%	0.094
Positive Predictive Value (95% CI)	2.0	(0.6,4.6)	13.4	(10.2,17.2)	7.3	(5.0,10.2)	29.0	(27.2,30.9)	40.7	(36.2,45.2)							
Negative Predictive Value (95% CI)	98.2	(95.9,99.4)	96.6	(93.1,98.6)	96.3	(94.2,97.8)	79.9	(77.1,82.4)	93.8	(92.0,95.3)							
Inflation Factor and "Bias"	25.0	large	6.6	large	8.7	large	2.7	large	1.9	large							
AUC and "accuracy"	0.5	low	0.6	moderate	0.6	low	0.6	low	0.8	high							
**Survey reported - Vaginal births**																	
Observer coverage %	2.3	(1.1,5.1)	14.0	(11.0,17.6)	5.6	(4.2,7.4)	27.2	(25.7,28.6)	33.2	(30.3,36.2)	14.4	(5.4,26.7)	442.3	4	0.000	99.1%	0.121
Survey reported coverage %	59.9	(56.4,63.4)	85.3	(83.3,87.1)	61.1	(59.8,62.3)	73.5	(72.3,74.7)	65.1	(62.2,68.0)	69.5	(60.5,77.9)	446.6	4	0.000	99.1%	0.046
"Don't know" responses %	0.1	(0.0,1.0)	0.3	(0.1,0.8)	0.3	(0.2,0.4)	0.8	(0.6,1.1)	2.6	(1.8,3.7)	0.6	(0.2,1.3)	53.8	4	0.000	92.6%	0.005
**Current survey - Count "don't know" as "no"**																	

Sensitivity % (95% CI)	†	†	88.3	(77.4,95.2)	71.4	(55.4,84.3)	78.1	(75.2,80.8)	85.6	(80.4,89.9)	82.5	(76.4,88.0)	11.4	4	0.023	64.8%	0.014
Specificity % (95% CI)	†	†	19.7	(15.7,24.2)	48.1	(44.4,51.8)	27.8	(25.9,29.6)	41.5	(37.0,46.0)	35.9	(25.8,46.7)	159.9	4	0.000	97.5%	0.061
Percent agreement (TP+TN/n) %	45.7	(39.6,52.0)	29.6	(25.4,34.1)	49.4	(45.8,53.0)	41.6	(39.9,43.3)	55.7	(52.0,59.3)	44.4	(36.9,51.9)	92.9	4	0.000	95.7%	0.028
Positive Predictive Value % (95% CI)	†	†	15.6	(11.9,19.9)	7.5	(5.1,10.5)	29.1	(27.3,31.0)	41.1	(36.6,45.7)							
Negative Predictive Value % (95% CI)	†	†	90.9	(82.2,96.3)	96.6	(94.2,98.2)	76.9	(73.9,79.7)	85.8	(80.6,90.0)							
Inflation Factor and "Bias"	†	†	5.7	large	9.6	large	2.7	large	2.1	large							
AUC and "accuracy"	†	†	0.5	low	0.6	low	0.5	low	0.6	moderate							
**Consider only "yes" and "no" (exclude don't know)**																	
Sensitivity (95% CI)	†	†	89.8	(79.2,96.2)	71.4	(55.4,84.3)	78.5	(75.6,81.2)	86.7	(81.6,90.9)	83.4	(76.6,89.3)	13.9	4	0.007	71.3%	0.019
Specificity (95% CI)	†	†	19.4	(15.4,23.9)	48.0	(44.3,51.8)	27.2	(25.4,29.1)	40.0	(35.5,44.6)	35.4	(25.2,46.2)	160.5	4	0.000	97.5%	0.061
Percent agreement (TP+TN/n) %	45.5	(39.4,51.8)	29.5	(25.3,34.0)	49.3	(45.8,52.9)	41.4	(39.7,43.1)	55.2	(51.5,58.9)	44.1	(36.7,51.6)	90.0	4	0.000	95.6%	0.027
Positive Predictive Value (95% CI)	†	†	15.6	(11.9,19.9)	7.5	(5.1,10.5)	29.1	(27.3,31.0)	41.1	(36.6,45.7)							
Negative Predictive Value (95% CI)	†	†	92.0	(83.4,97.0)	96.6	(94.2,98.2)	76.9	(73.8,79.8)	86.2	(80.9,90.5)							
Inflation Factor and "Bias"	†	†	5.7	large	9.5	large	2.7	large	2.1	large							
AUC and "accuracy"	†	†	0.6	low	0.6	low	0.5	low	0.6	moderate							
**Survey reported - Caesarean**																	
Observer coverage %	1.4	(0.5,3.6)	0.5	(0.1,3.6)	3.5	(1.4,8.1)	3.6	(1.7,7.4)	3.6	(2.6,5.1)	2.4	(1.2,3.9)	10.7	4	0.030	62.6%	0.006
Survey reported coverage %	46.2	(44.1,48.4)	39.1	(36.0,42.2)	9.8	(8.1,11.8)	6.8	(4.5,10.2)	1.5	(1.0,2.3)	17.1	(2.3,41.3)	1686.2	4	0.000	99.8%	0.382
"Don't know" responses %	#VALUE!	(0.0,0.0)	0.1	(0.0,0.7)	0.6	(0.3,1.4)	#VALUE!	(0.0,0.0)	2.5	(1.8,3.4)	0.4	(0.0,1.5)	85.4	4	0.000	95.3%	0.018
**Current survey - Count "don't know" as "no"**																	
Sensitivity % (95% CI)	†	†	†	†	†	†	†	†	0.0	(0.0,14.2)	0.0	(0.0,2.6)	3.7	4	0.445	0.0%	0.000
Specificity % (95% CI)	†	†	†	†	†	†	†	†	99.2	(98.2,99.7)	85.3	(62.6,98.5)	344.2	4	0.000	98.8%	0.355
Percent agreement (TP+TN/n) %	58.4	(52.6,64.0)	69.5	(62.5,75.7)	86.4	(79.0,91.5)	90.3	(84.2,94.2)	95.7	(93.9,97.0)	82.0	(63.2,95.1)	229.4	4	0.000	98.3%	0.230
Positive Predictive Value % (95% CI)	†	†	†	†	†	†	†	†	0.0	(0.0,52.2)							
Negative Predictive Value % (95% CI)	†	†	†	†	†	†	†	†	96.4	(94.7,97.7)							
Inflation Factor and "Bias"	†	†	†	†	†	†	†	†	0.2	large							
AUC and "accuracy"	†	†	†	†	†	†	†	†	0.5	low							
**Consider only "yes" and "no" (exclude don't know)**																	
Sensitivity (95% CI)	†	†	†	†	†	†	†	†	0.0	(0.0,14.2)	0.0	(0.0,2.6)	3.7	4	0.445	0.0%	0.000
Specificity (95% CI)	†	†	†	†	†	†	†	†	99.2	(98.2,99.7)	85.3	(62.7,98.5)	339.2	4	0.000	98.8%	0.352
Percent agreement (TP+TN/n) %	58.4	(52.6,64.0)	69.5	(62.5,75.7)	86.4	(79.0,91.5)	90.3	(84.2,94.2)	95.6	(93.7,96.9)	82.0	(63.3,95.0)	224.7	4	0.000	98.2%	0.226
Positive Predictive Value (95% CI)	†	†	†	†	†	†	†	†	0.0	(0.0,52.2)							
Negative Predictive Value (95% CI)	†	†	†	†	†	†	†	†	96.3	(94.6,97.6)							
Inflation Factor and "Bias"	†	†	†	†	†	†	†	†	0.2	large							

AUC and "accuracy"	†	†	†	†	†	†	†	†	0.5	low	

† =result suppressed due to 10 or fewer count per column of two-by-two table
N= 7,802 babies observed for ≥1 hour after birth [1]

References:

1. Day L, Rahman QS, Rahman A, Salim N, KC A, Ruysen H, Tahsina T, Masanja H, Basnet O, Gore-langton G *et al*: Assessment of the validity of the measurement of newborn and maternal health-care coverage in hospitals (EN-BIRTH): an observational study. *Lancet Global* [2020] doi: 10.1016/S2214-109X(20)30504-0.
