# Supplementary material for: Immediate newborn care and breastfeeding: EN-BIRTH multi-country validation study
Source: BMC Pregnancy Childbirth. 2021 Mar 26;21(Suppl 1):237. doi: 10.1186/s12884-020-03421-w (PMC7995709; doi:10.1186/s12884-020-03421-w)
Supplement: Supplementary file 11 — Additional File 11. Individual-level validation of register recording for early initiation of breastfeeding, EN-BIRTH study (n = 7802). [file 12884_2020_3421_MOESM11_ESM.pdf]

Every Newborn BIRTH multi-country validation study: informing measurement of coverage and quality of maternal and newborn care

Immediate newborn care and breastfeeding: EN-BIRTH multi-country validation study

Additional File 11: Individual-level validation of register recording for early initiation of breastfeeding, EN-BIRTH study (n=7,802)

	Bangladesh				Nepal		Tanzania				All sites							
	Azimpur Tertiary		Kushtia District		Pokhara Regional		Temeke Regional		Muhimbili National									
**Register recorded - All mode**	%	95%CI	%	95%CI			%	95%CI	%	95%CI	Pooled Random Effects		Q	df	p-val.	i²	τ²
Observer coverage %	1.8	(1.0,3.3)	9.8	(7.7,12.4)	0.0	(0.0,0.0)	26.0	(24.6,27.4)	19.1	(17.3,20.9)	12.5	(4.6,23.6)	363.3	3	0.000	99.2%	0.087
Register recorded coverage %	91.7	(90.5,92.8)	96.8	(95.9,97.5)	0.0	(0.0,0.0)	95.3	(94.7,95.7)	43.8	(42.1,45.5)	85.9	(58.1,99.6)	4029.1	3	0.000	99.9%	0.423
Not recorded and not possible to record %	7.8	(6.8,9.0)	3.1	(2.4,4.0)	0.0	(0.0,0.0)	2.3	(1.9,2.6)	23.5	(22.1,24.9)	7.6	(1.1,19.2)	1192.6	3	0.000	99.7%	0.125
Not readable %	0.0	(0.0,0.2)	0.0	(0.0,0.2)	0.0	(0.0,0.0)	0.1	(0.1,0.3)	0.2	(0.1,0.4)	0.1	(0.0,0.2)	11.1	3	0.011	73.0%	0.001
**Current HMIS - count "not recorded" as "no"**																	
Sensitivity % (95% CI)	†	†	98.2	(90.6,100.0)	†	†	97.3	(96.1,98.2)	64.5	(59.2,69.5)	93.2	(68.7,100.0)	233.3	3	0.000	98.7%	0.375
Specificity % (95% CI)	†	†	2.8	(1.6,4.7)	†	†	4.9	(4.2,5.8)	55.4	(52.8,58.0)	13.0	(0.0,43.5)	1616.6	3	0.000	99.8%	0.508
Percent agreement (TP+TN/n) %	7.7	(5.5,10.5)	12.7	(10.2,15.8)	0.0	(0.0,0.0)	29.0	(27.6,30.5)	57.2	(54.9,59.4)	24.6	(8.5,45.7)	790.3	3	0.000	99.6%	0.206
Positive Predictive Value % (95% CI)	†	†	10.4	(8.0,13.4)	†	†	26.5	(25.1,28.0)	25.5	(22.6,28.5)							
Negative Predictive Value % (95% CI)	†	†	93.3	(68.1,99.8)	†	†	84.0	(77.4,89.2)	86.8	(84.5,88.9)							
Inflation Factor and "Bias"	†	†	9.4	large	†	†	3.7	large	2.5	large							
AUC and "accuracy"	†	†	0.5	low	†	†	0.5	low	0.6	low							
**Consider only "given" and "not given" (exclude not recorded)**																	
Sensitivity % (95% CI)	†	†	100.0	(93.6,100.0)	†	†	99.3	(98.5,99.7)	81.7	(76.6,86.1)	97.6	(83.9,100.0)	109.3	3	0.000	97.3%	0.201
Specificity % (95% CI)	†	†	0.2	(0.0,1.2)	†	†	2.6	(2.0,3.3)	43.0	(40.1,45.9)	6.4	(0.0,29.2)	1171.6	3	0.000	99.7%	0.412
Percent agreement (TP+TN/n) %	2.8	(1.6,4.9)	10.6	(8.3,13.5)	0.0	(0.0,0.0)	27.9	(26.5,29.4)	50.5	(47.9,53.1)	20.0	(5.9,39.6)	663.8	3	0.000	99.5%	0.194
Positive Predictive Value % (95% CI)	†	†	10.4	(8.0,13.4)	†	†	26.5	(25.1,28.0)	25.5	(22.6,28.5)							
Negative Predictive Value % (95% CI)	†	†	100.0	(2.5,100.0)	†	†	90.9	(82.2,96.3)	90.8	(88.0,93.1)							
Inflation Factor and "Bias"	†	†	9.6	large	†	†	3.7	large	3.2	large							
AUC and "accuracy"	†	†	0.5	low	†	†	0.5	low	0.6	moderate							
**Register recorded - Vaginal Births**																	
Observer coverage %	2.3	(1.1,5.1)	14.0	(11.0,17.6)	0.0	(0.0,0.0)	27.2	(25.7,28.6)	33.2	(30.3,36.2)	17.3	(8.0,29.1)	210.8	3	0.000	98.6%	0.081
Register recorded coverage (%)	97.7	(96.0,98.6)	95.8	(94.5,96.9)	0.0	(0.0,0.0)	96.4	(95.9,96.9)	64.1	(61.6,66.5)	91.4	(74.9,99.5)	1008.2	3	0.000	99.7%	0.211
Not recorded and not possible to record %	1.4	(0.7,2.8)	4.0	(3.0,5.3)	0.0	(0.0,0.0)	1.9	(1.6,2.3)	20.2	(18.2,22.3)	5.3	(0.5,14.7)	517.9	3	0.000	99.4%	0.108
Not readable %	0.0	(0.0,0.7)	0.0	(0.0,0.3)	0.0	(0.0,0.0)	0.1	(0.1,0.2)	0.1	(0.0,0.5)	0.1	(0.0,0.1)	1.8	3	0.616	0.0%	0.000

**Current HMIS - count "not recorded" as "no"**																	
Sensitivity % (95% CI)	†	†	98.2	(90.6,100.0)	†	†	97.5	(96.3,98.4)	67.8	(62.4,72.9)	93.8	(70.7,100.0)	190.9	3	0.000	98.4%	0.325
Specificity % (95% CI)	†	†	3.1	(1.5,5.6)	†	†	3.6	(2.9,4.4)	37.6	(33.8,41.4)	8.9	(0.2,27.5)	468.6	3	0.000	99.4%	0.256
Percent agreement (TP+TN/n) %	5.9	(3.4,10.1)	17.2	(13.8,21.4)	0.0	(0.0,0.0)	29.2	(27.7,30.7)	47.6	(44.4,50.8)	23.4	(11.5,38.0)	250.4	3	0.000	98.8%	0.103
Positive Predictive Value % (95% CI)	†	†	15.1	(11.6,19.1)	†	†	27.4	(26.0,29.0)	35.0	(31.2,38.9)							
Negative Predictive Value % (95% CI)	†	†	90.9	(58.7,99.8)	†	†	79.5	(71.0,86.4)	70.2	(65.0,75.0)							
Inflation Factor and "Bias"	†	†	6.5	large	†	†	3.6	large	1.9	large							
AUC and "accuracy"	†	†	0.5	low	†	†	0.5	low	0.5	low							
**Consider only "given" and "not given" (exclude not recorded)**																	
Sensitivity % (95% CI)	†	†	100.0	(93.6,100.0)	†	†	99.4	(98.6,99.8)	85.3	(80.3,89.4)	98.4	(86.8,100.0)	79.3	3	0.000	96.2%	0.153
Specificity % (95% CI)	†	†	0.3	(0.0,1.7)	†	†	1.7	(1.3,2.3)	21.8	(18.3,25.6)	4.1	(0.0,14.4)	231.1	3	0.000	98.7%	0.139
Percent agreement (TP+TN/n) %	4.5	(2.4,8.4)	15.3	(12.0,19.3)	0.0	(0.0,0.0)	28.3	(26.8,29.8)	42.8	(39.3,46.3)	21.0	(10.2,34.5)	201.5	3	0.000	98.5%	0.093
Positive Predictive Value % (95% CI)	†	†	15.1	(11.6,19.1)	†	†	27.4	(26.0,29.0)	35.0	(31.2,38.9)							
Negative Predictive Value % (95% CI)	†	†	100.0	(2.5,100.0)	†	†	88.0	(75.7,95.5)	75.0	(67.2,81.7)							
Inflation Factor and "Bias"	†	†	6.6	large	†	†	3.6	large	2.4	large							
AUC and "accuracy"	†	†	0.5	low	†	†	0.5	low	0.5	low							
**Register recorded - Caesarean**																	
Observer coverage (%)	1.4	(0.5,3.6)	0.5	(0.1,3.6)	0.0	(0.0,0.0)	3.6	(1.7,7.4)	3.6	(2.6,5.1)	2.2	(0.9,4.0)	10.3	3	0.016	70.8%	0.008
Register recorded coverage (%)	89.7	(88.1,91.0)	98.2	(97.0,99.0)	0.0	(0.0,0.0)	79.7	(75.7,83.2)	28.6	(26.7,30.7)	78.3	(37.8,99.7)	2459.2	3	0.000	99.9%	0.747
Not recorded and not possible to record	10.0	(8.6,11.5)	1.8	(1.0,3.0)	0.0	(0.0,0.0)	6.9	(4.9,9.7)	25.9	(24.0,27.9)	9.6	(2.0,22.0)	407.9	3	0.000	99.3%	0.123
Not readable	0.0	(0.0,0.2)	0.0	(0.0,0.5)	0.0	(0.0,0.0)	0.4	(0.1,1.8)	0.3	(0.1,0.7)	0.1	(0.0,0.4)	10.9	3	0.012	72.5%	0.002
**Current HMIS - count "not recorded" as "no"**																	
Sensitivity (95% CI)	†	†	†	†	†	†	†	†	27.6	(12.7,47.2)	63.0	(15.7,99.3)	9.8	2	0.007	79.6%	0.451
Specificity (95% CI)	†	†	†	†	†	†	†	†	69.4	(66.1,72.5)	21.9	(0.2,63.0)	644.9	3	0.000	99.5%	0.759
Percent agreement (TN+TP/n) all observed	9.1	(6.1,13.5)	2.4	(0.9,6.2)	0.0	(0.0,0.0)	25.8	(20.0,32.6)	68.0	(64.7,71.0)	22.4	(0.5,62.0)	607.4	3	0.000	99.5%	0.700
Positive Predictive Value (95% CI)	†	†	†	†	†	†	†	†	48.0	(40.0,57.0)							
Negative Predictive Value (95% CI)	†	†	†	†	†	†	†	†	3.1	(1.3,6.0)							
Inflation Factor and "Bias"	†	†	†	†	†	†	†	†	9.0	large							
AUC and "accuracy"	†	†	†	†	†	†	†	†	1.0	high							
**Consider only "given" and "not given" (exclude not recorded)**																	
Sensitivity (95% CI)	†	†	†	†	†	†	†	†	38.1	(18.1,61.6)	71.5	(26.5,100.0)	7.2	2	0.027	72.3%	0.346
Specificity (95% CI)	†	†	†	†	†	†	†	†	60.2	(56.3,64.0)	11.2	(0.0,53.4)	706.8	3	0.000	99.6%	0.934
Percent agreement (TN+TP/n) 2x2 table only	1.4	(0.4,4.1)	0.0	(0.0,2.2)	0.0	(0.0,0.0)	18.5	(13.3,25.1)	59.5	(55.7,63.2)	13.1	(0.0,53.0)	624.4	3	0.000	99.5%	0.808
Positive Predictive Value (95% CI)	†	†	†	†	†	†	†	†	3.1	(1.3,6.0)							

Negative Predictive Value (95% CI)	†	†	†	†	†	†	†	†	96.7	(94.4,98.2)	
Inflation Factor and "Bias"	†	†	†	†	†	†	†	†	12.4	large	
AUC and "accuracy"	†	†	†	†	†	†	†	†	0.5	low	

N/A=data element not captured by routine register
† =result suppressed due to 10 or fewer count per column of two-by-two table
N= 7,802 babies observed for ≥1 hour after birth [1]

References:

1. Day L, Rahman QS, Rahman A, Salim N, KC A, Ruysen H, Tahsina T, Masanja H, Basnet O, Gore-langton G *et al*: Assessment of the validity of the measurement of newborn and maternal health-care coverage in hospitals (EN-BIRTH): an observational study. *Lancet Global* [2020] doi: 10.1016/S2214-109X(20)30504-0.
