# Supplementary material for: Immediate newborn care and breastfeeding: EN-BIRTH multi-country validation study
Source: BMC Pregnancy Childbirth. 2021 Mar 26;21(Suppl 1):237. doi: 10.1186/s12884-020-03421-w (PMC7995709; doi:10.1186/s12884-020-03421-w)
Supplement: Supplementary file 12 — Additional File 12. Characteristics of women observed in labour and delivery wards for < 1 h, EN-BIRTH study (n = 12,554). [file 12884_2020_3421_MOESM12_ESM.pdf]

Every Newborn BIRTH multi-country validation study: informing measurement of coverage and quality of maternal and newborn care

Immediate newborn care and breastfeeding: EN-BIRTH multi-country validation study

Additional File 12: Characteristics of women observed in labour and delivery wards for <1 hour, EN-BIRTH study (n=12,554)

	Bangladesh		Nepal	Tanzania		Total
	**Azimpur Tertiary**	**Kushtia District**	**Pokhara Regional**	**Temeke Regional**	**Muhimbili National**	
	n(%)	n(%)	n(%)	n(%)	n(%)	n(%)
Total	2300(100)	1623(100)	6143(100)	1842(100)	646(100)	12554(100)
**Woman's Age**						
<18 years	20(0.9)	1(0.1)	265(4.3)	9(0.5)	1(0.2)	296(2.4)
18-19 years	364(15.8)	134(8.3)	673(11)	209(11.3)	19(2.9)	1399(11.1)
20-24 years	918(39.9)	611(37.6)	2565(41.8)	620(33.7)	135(20.9)	4849(38.6)
25-29 years	704(30.6)	527(32.5)	1787(29.1)	475(25.8)	194(30)	3687(29.4)
30-34 years	227(9.9)	246(15.2)	672(10.9)	298(16.2)	177(27.4)	1620(12.9)
35+ years	67(2.9)	104(6.4)	181(2.9)	231(12.5)	120(18.6)	703(5.6)
**Woman's education**						
No Education	30(1.3)	48(3)	231(3.8)	52(2.8)	16(2.5)	377(3)
Primary incomplete	86(3.7)	89(5.5)	208(3.4)	20(1.1)	8(1.2)	411(3.3)
Primary complete	253(11)	228(14)	239(3.9)	8(0.4)	0(0)	728(5.8)
Secondary incomplete	782(34)	650(40)	1380(22.5)	1099(59.7)	226(35)	4137(33)
Secondary complete	1017(44.2)	582(35.9)	3742(60.9)	651(35.3)	395(61.1)	6387(50.9)
Don't know	132(5.7)	26(1.6)	343(5.6)	12(0.7)	1(0.2)	514(4.1)
**Gestational age at admission (weeks)**						
<28 weeks	0(0)	1(0.1)	6(0.1)	0(0)	1(0.2)	8(0.1)
28-31 weeks	1(0)	19(1.2)	18(0.3)	9(0.5)	25(3.9)	72(0.6)
32-36 weeks	520(22.6)	316(19.5)	262(4.3)	381(20.7)	162(25.1)	1641(13.1)
37+ weeks	1779(77.3)	1287(79.3)	5857(95.3)	1452(78.8)	458(70.9)	10833(86.3)
**Mode of birth**						
Vaginal birth	495(21.5)	867(53.4)	5255(85.5)	1714(93.1)	143(22.1)	8474(67.5)
Caesarean section	1804(78.4)	756(46.6)	888(14.5)	128(6.9)	503(77.9)	4079(32.5)
Missing	1(0)	0(0)	0(0)	0(0)	0(0)	1(0)

N= 12,554 women observed for less than 1 hour with a live birth
