# Supplementary material for: Immediate newborn care and breastfeeding: EN-BIRTH multi-country validation study
Source: BMC Pregnancy Childbirth. 2021 Mar 26;21(Suppl 1):237. doi: 10.1186/s12884-020-03421-w (PMC7995709; doi:10.1186/s12884-020-03421-w)

Every Newborn BIRTH multi-country validation study: informing measurement of coverage and quality of maternal and newborn care

Immediate newborn care and breastfeeding: EN-BIRTH multi-country validation study

Additional File 14: Register recording order and prioritisation for breastfeeding, EN-BIRTH study

To identify how health care provision and labour ward register documentation relate to one another on labour ward, we designed a third tool called the "care-to-documentation checklist". The tool captured the process, flow and sequence of recording data in registers by selected indicators:

- Which health worker cadre usually/ sometimes provides the care?
- Which cadre records the care?
- What is the order of documentation in labour ward documents (among register, patient notes, drug charts, partograph)?
- What is the estimated time in minutes between intervention given and documentation?

These close-ended questions were asked by the researcher to respondents, immediately after their in depth interview (but not to FGD respondents) and relate to findings show in Additional files 11 & 12.

a. As reported by EN-BIRTH data collectors

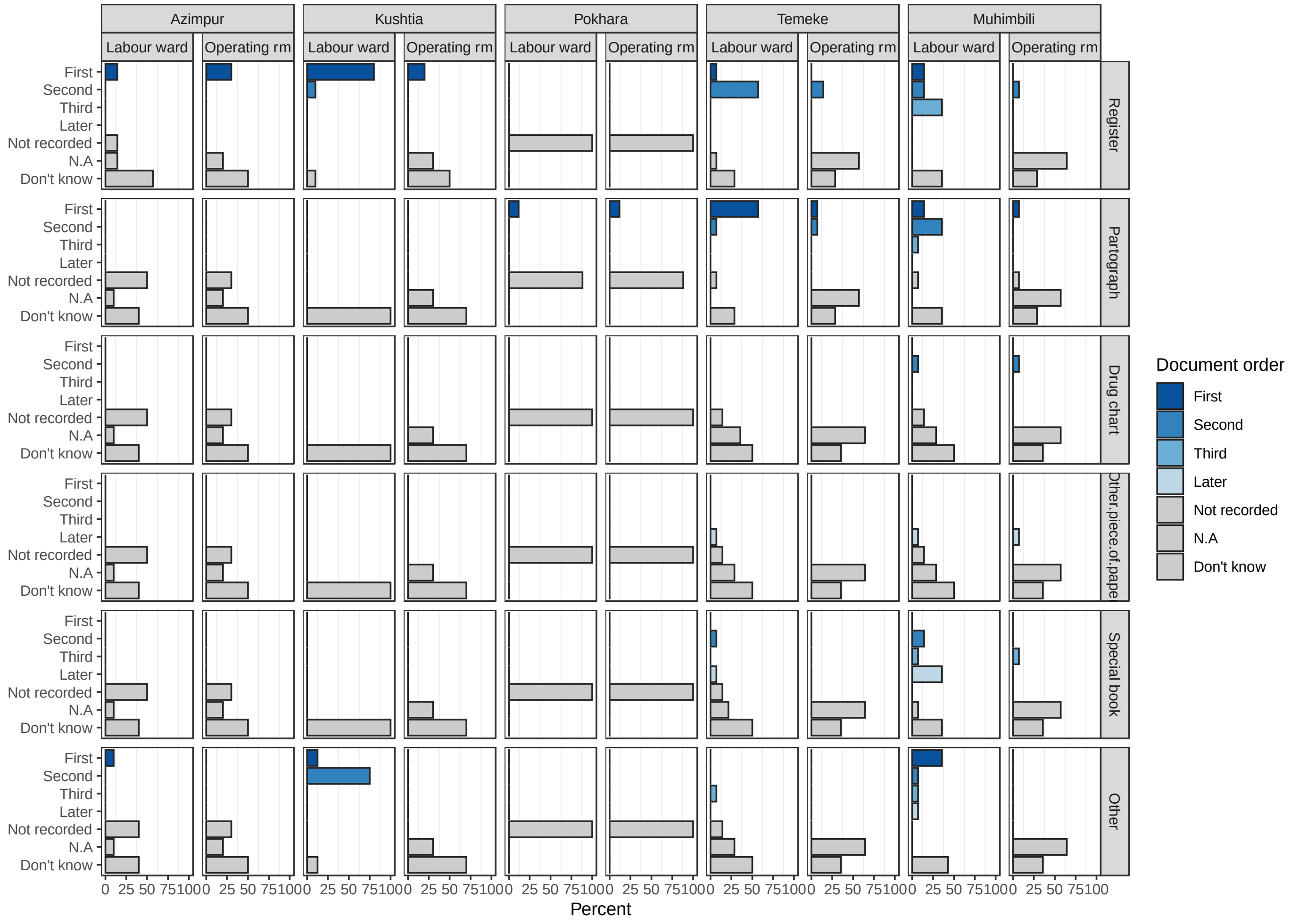

b. As reported by health workers

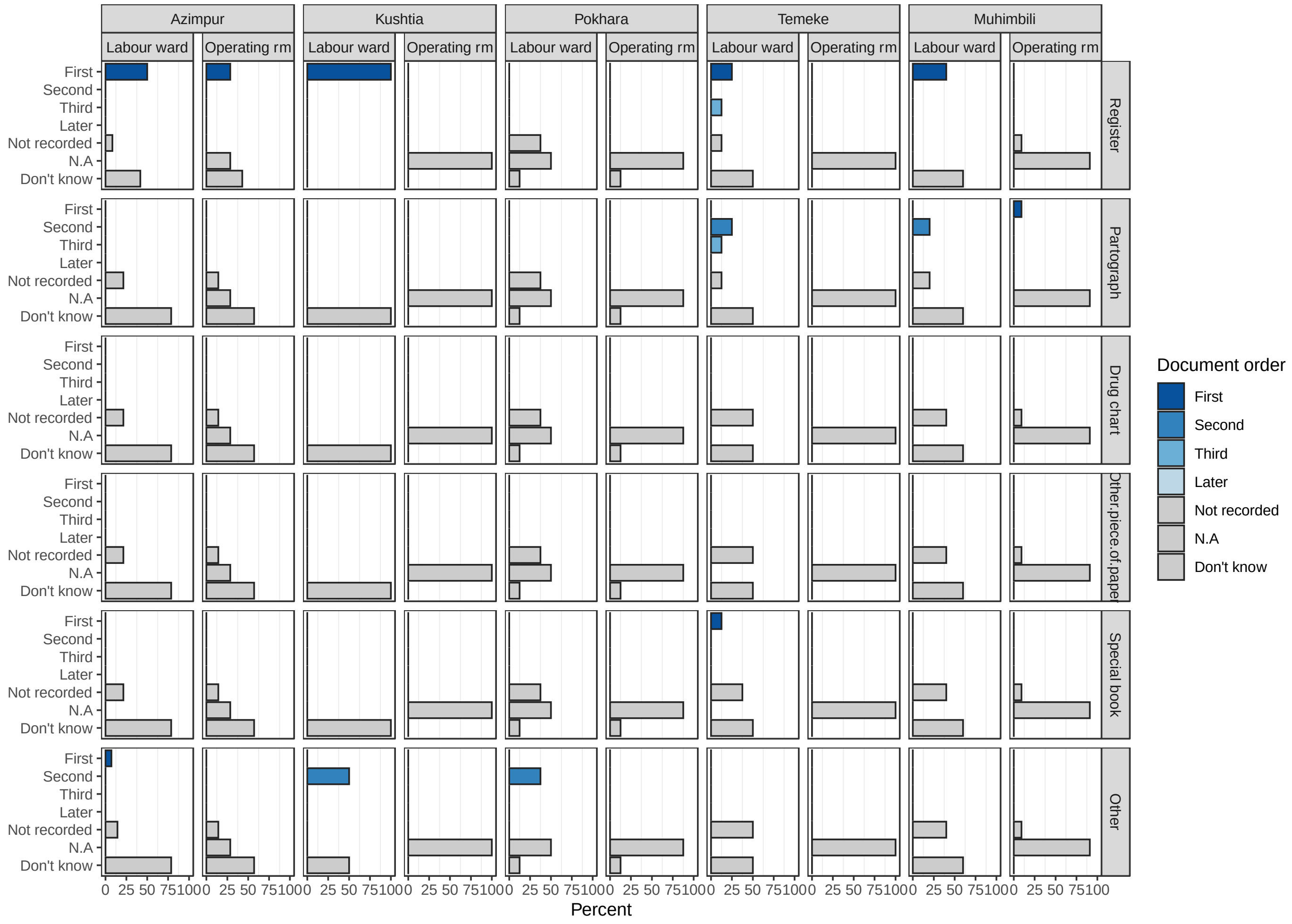

Supplement: Supplementary file 14 — Additional File 14. Register recording order and prioritisation for breastfeeding, EN-BIRTH study. [file 12884_2020_3421_MOESM14_ESM.pdf]
