# Supplementary material for: Immediate newborn care and breastfeeding: EN-BIRTH multi-country validation study
Source: BMC Pregnancy Childbirth. 2021 Mar 26;21(Suppl 1):237. doi: 10.1186/s12884-020-03421-w (PMC7995709; doi:10.1186/s12884-020-03421-w)
Supplement: Supplementary file 15 — Additional File 15. Inter-observer agreement for early initiation of breastfeeding using Kappa, EN-BIRTH study. [file 12884_2020_3421_MOESM15_ESM.pdf]

Every Newborn BIRTH multi-country validation study: informing measurement of coverage and quality of maternal and newborn care

Immediate Newborn Care and Breastfeeding Practices: EN-BIRTH Multi-Country Study

Additional File 15: Inter-observer agreement for early initiation of breastfeeding using Kappa, EN-BIRTH study

		**Bangladesh**		**Nepal**	**Tanzania**	
		**Azimpur Tertiary**	**Kushtia District**	**Pokhara Regional**	**Temeke Regional**	**Muhimbili National**
**Breastfeeding**		Kappa	Kappa	Kappa	Kappa	Kappa
L&D Observation	n=862	1.000	0.791	0.526	0.554	0.762
L&D Data Extraction	n=2120	1.000	0.000		0.259	-0.030

Kappa agreement cut offs: <0.71 considered high/substantial disagreement for observation, and <0.9 considered high/substantial disagreement for data extraction, Day et al [1]

References

1. Day L, Rahman QS, Rahman A, Salim N, KC A, Ruysen H, Tahsina T, Masanja H, Basnet O, Gore-langton G *et al*: Assessment of the validity of the measurement of newborn and maternal health-care coverage in hospitals (EN-BIRTH): an observational study. *Lancet Global* [2020] doi: 10.1016/S2214-109X(20)30504-0.
